# Experimental IR, Raman, and UV-Vis Spectra DFT Structural and Conformational Studies: Bioactivity and Solvent Effect on Molecular Properties of Methyl-Eugenol

**DOI:** 10.3390/molecules28145409

**Published:** 2023-07-14

**Authors:** Rohit Kumar Yadav, Bhoopendra Yadav, R. A. Yadav, Irena Kostova

**Affiliations:** 1 Department of Physics, Institute of Science, Banaras Hindu University, Varanasi 221005, India; rohit.yadav@bhu.ac.in (R.K.Y.); bhoopendra.yadav@bhu.ac.in (B.Y.); 2 Department of Chemistry, Faculty of Pharmacy, Medical University, 2 Dunav St., 1000 Sofia, Bulgaria

**Keywords:** structural, conformational and vibrational studies, MEP, HOMO-LUMO analysis, IR, Raman and UV-vis spectra, barrier heights, bioactivity

## Abstract

**Highlights:**

**What are the main findings?**

- The ME molecule has 21 stable configurations.
- For all the tops (except =CH_2_), the barrier heights are of the same order, while the =CH_2_ top has a barrier height one order of magnitude higher.
- Like estragole and eugenol, ME also has the same Fermi doublets for the following modes: ν_s_(–CH_2_) and 2 × β_s_(–CH_2_); ν_s_(CH_3_) and 2 × δ_s_(CH_3_).
- The ME molecule has three active sites.
- Vibrational analysis suggests that the solvents affect the internal modes of both OCH_3_ moieties strongly.

**What is the implication of the main finding?**

- The methyl-eugenol molecule could be a good choice for the pharmacological applications
- The OCH_3_ moieties of methyl-eugenol play significant role in interaction with other molecules.

**Abstract:**

Structural, conformational, and spectroscopic investigations of methyl-eugenol were made theoretically at the B3LYP-6-311++G**level. Experimental IR, Raman, and UV-vis spectra were investigated and analyzed in light of the computed quantities. Conformational analysis was carried out with the help of total energy vs. dihedral angle curves for different tops, yielding 21 stable conformers, out of which only two have energies below the room temperature relative to the lowest energy conformer. The effect of the solvent on different molecular characteristics was investigated theoretically. MEP and HOMO-LUMO analysis were carried out and barrier heights and bioactivity scores were determined. The present investigation suggests that the molecule has three active sites with moderate bioactivity. The solvent–solute interaction is found to be dominant in the vicinity of the methoxy moieties.

## 1. Introduction

Methyl-eugenol (ME) is a member of the family of phenylpropenes. A total of ~450 plant species from across 80 families were found to contain ME in essential oils from plant leaves, roots, stems, flowers, or whole plant extracts [1]. The oils constituting more than 0.1% of methyl eugenol are calamus, rose, tea tree oil, green myrtle, citronella, lemon balm, camphor oil. Common herbs and spices containing ME are basils [2], lemongrass, bay leaves, cloves, tarragon, allspice, nutmeg, and mace [3,4]. ME is also present in fruits such as banana, grapefruit, and some other forest fruits. It is a natural product and bears strong potential in medicinal and agriculture areas like aromatherapy, massage, and liver injury [5], for its anti-cancer [6], anti-allergic [7], and anti-oxidative effects [8], as an anesthetic agent for rodents [9], in insecticides, and for its antifungal and antibacterial actions [10], etc. It has flavor and fragrance properties occurring naturally in various plants including some herbs, distinct food resources, and essential oils. Natural ME is used as a flavoring agent in food and as a fragrance in cosmetic products [11]; synthesized ME is used as an insect attractant but not used as a flavoring agent in food and fragrance in cosmetic products as it may cause cancer in humans/animals [12,13]. It is also used in low/high concentration for fruit fly attractant/replant [14]. It is a safe anesthetic agent as well as anti-depressive and reduces anxiety level for rats [9,15] because of its capability of inducing partial or total loss of sensation. Rietjens et al. [16] studied the metabolic and toxic behaviors of ME in different orientation of the functional groups.

Structural and vibrational investigations of ME were carried out by Chowdhry et al. [17], in which they considered only the three lower energy conformers. The vibrational assignments proposed by these authors are also doubtful in several cases. In the present paper, structural and spectroscopic investigations were carried out for the ME molecule at the level B3LYP-6-311++G**, in addition to the experimental IR, Raman, and UV-vis spectral studies. The structural and vibrational computations were carried out also with water and ethanol as solvents. We have determined the barrier height, MEP, HOMO-LUMO analysis, and bioactive scores. To determine the total number of possible conformers, we scanned total energy vs. dihedral angle curves for different tops. A total of 21 pairs of stable conformers were found. In each pair, one conformer is the enantiomer of the other conformer. Out of these 21 conformers, only 2 conformers are found to exist below 300 K relative to the lowest energy conformers, as also reported by earlier workers [17].

## 2. Result and Discussion

**Figure 1:**
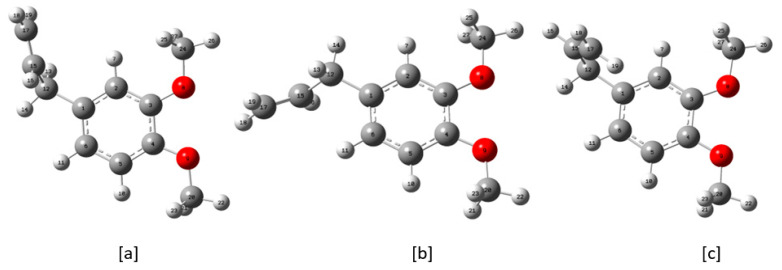
(**a**–**c**) Front view of the three lower energy conformers of ME.

The optimized structure of the ME molecule is shown in Figure 1 (C-I). There are seven tops in ME molecule. By scanning the total energy vs. dihedral angles surfaces, we search the total number of possible conformers.

### 2.1. Determination of Conformers

To search the total number of possible conformers of ME, we scanned the total energy vs. dihedral angle curves for different tops. The rotations of the two CH_3_ tops about their respective O-C axes do not yield any new structures. However, each of the rotations of the OCH_3_ tops about their respective C-O axes gives rise to 3-fold potential barrier, yielding only nine structures, out of which two structures are not stable due to strong steric hindrance. Therefore, the two OCH_3_ tops on rotating would yield seven possible stable structures. Similarly, the rotation of the =CH_2_ top about the C=C axis does not produce a new structure. However, rotations of the –CH=CH_2_ and –CH_2_–CH=CH_2_ tops about their respective axes result in 3- and 2-fold potential barriers, giving rise to six possible different structures. Therefore, the total number of possible stable structures comes out to be 7 × 6 = 42. Out of these 42 possible conformers, there are 21 pairs with different energies and in each pair one structure is the mirror of the other structure. One structure of such a pair is called the enantiomer of the other structure. The total energy and their energy differences with respect to the lowest energy conformer are listed in Table 1. The first three lower energy conformers of the ME molecule are shown in Figure 1. The conformers C-II and C-III are at higher energies by 215 and 312 K, respectively, above the conformer C-I.

According to the orientations of the three functional groups, the 21 conformers could be classified into two categories: (i) conformers with the same allyl group orientation and (ii) conformers with the same methoxy groups orientations. According to the allyl group orientations, the conformers could be divided into three categories, each of which contains seven conformers. Similarly, according to the methoxy groups orientations the conformers could be divided into seven categories, each of which contains three conformers (Table S1). The calculated relative abundances of all the 21 conformers were listed in Table 1. For the conformer C-I, C-II, and C-III, the abundances are 32.1, 16.2, and 11.2, respectively. Assuming the presence of only these three conformers—C-I, C-II, and C-III—below room temperature, the relative abundances are 53.9, 27.2, and 18.9, respectively, with an approximate ratio of 6:3:2.

### 2.2. Molecular Geometries

The optimized geometrical parameters of the three lower energy conformers (C-I, C-II, and C-III) are collected in Table S2. In going from one low energy conformer to another, a few geometrical parameters are found to change considerably. The C_12_–C_15_/C_1_–C_12_ bond length is found to enhance/reduce by 0.006 Å/0.008 Å in conformer C-II. The phenyl ring C–C bond lengths show small changes in going from C-I to C-III. The largest C–C bond length is found to be C_1_–C_12_ (1.520 Å) for the three conformers. The largest C–H bond lengths were found for the C–H bonds of the methyl groups lying in the ring plane.

The bond angles α(C_2_–C_3_–O_8_) (124.8°) and α(C_5_–C_4_–O_9_) (125.1°) are found to be much larger and the bond angles α(C_4_–C_3_–O_8_) (115.6°) and α(C_3_–C_4_–O_9_) (115.1°) have much lower values as compared to the other α(C–C–H) and α(C–C–C) angles associated with phenyl ring moieties due to excess electrostatic repulsive force between the two O atoms. For the H atoms of the two methyl groups lying in the ring plane, the α(O–C–H) bond angle for both methoxy groups is found to be smaller (105.7) than the other two α(O–C–H) angles (111.5°).

### 2.3. Barrier Heights

**Figure 2:**
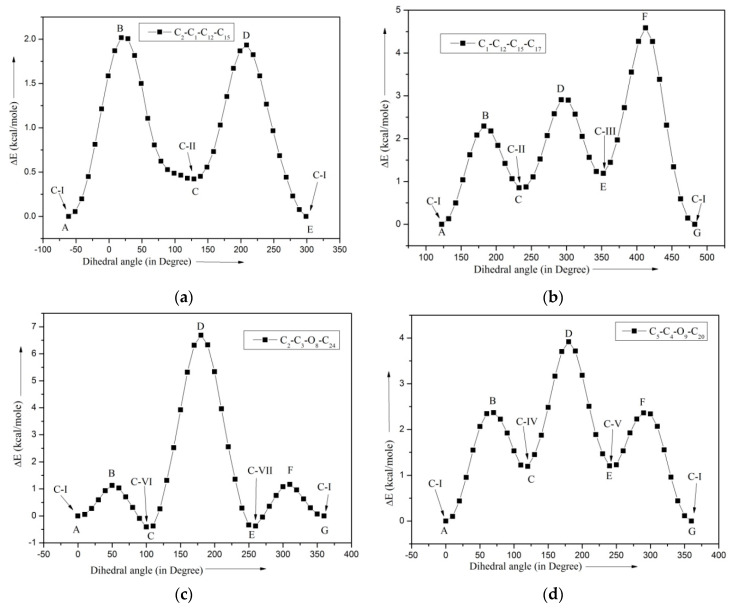
Total energy vs. dihedral angle curves for the tops (**a**)—CH_2_CHCH_2_, (**b**)—CHCH_2_, (**c**)—^8^OCH_3_, and (**d**)—^9^OCH_3_.

Referring to the Figure 1, the ME molecule has seven tops; namely, the two OCH_3_, two CH_3_, a CH_2_CHCH_2_, a CHCH_2_, and a =CH_2_ top. The total energy vs. dihedral angle curves for the four tops, which generate different conformers, are shown in Figure 2, and the three tops, which do not generate any new conformers, are shown in Figure S2. The number of minima in each curve gives the foldness of the corresponding barrier. The computed barrier heights are listed in Table 2. The rotations of the CH_3_ and =CH_2_ tops do not yield new structures. Each of the two CH_3_ tops has the barrier height of 3.34 kcal/mole, while the =CH_2_ top has the barrier height of 93.13 kcal/mole.

The remaining four tops CH_2_CHCH_2_, CHCH_2_, ^8^OCH_3_, and ^9^OCH_3_ are responsible for the formation of different conformers. The total energy vs. dihedral angle plot for the top CH_2_CHCH_2_ about the C_1_–C_12_ axis is shown in the Figure 2a, in which the points A and E correspond to the same configuration. The points A/E and C and, similarly, the points B and D are energetically different points. The transition A → C via B needs more energy than the transition E → C via D. The reverse transitions (C → A and C → E) follow a similar pattern but require less energy. This difference is a result of substituent OCH_3_ groups at the meta- and para-positions relative to the allyl group. Referring to Figure 2b, the points A/G, C, and E and similarly the points B, D, and F are energetically different points. The energies required for the transitions A → C, C → E, and E → G are found to be 2.36, 2.00, and 3.62 kcal/mole, respectively; for the reverse transitions, the required energies are 1.27, 1.98, and 4.73 kcal/mole, respectively. From Figure 2c, it could be seen that the points A/G and C/E and, similarly, the points B/F and D are energetically different points. The transitions A → C and G → E through the points B and F need the same amount (1.13 kcal/mole) of energy. For the lowest energy conformer, allyl moiety does not affect the barrier heights for the ^8^OCH_3_ and ^9^OCH_3_ tops. However, the transition C → E through the point D is found nearly six times higher than the energy required for the transitions A → C or G → E. Similarly, for Figure 2d, the local minima points A/G and C/E separated by the peak points B/F and D are energetically different points. The transitions from A → C and G → E through the points B and F are found to be ~2.36 kcal/mole, however, the transition C → E through the point D is found to be ~2.72 kcal/mole.

### 2.4. Bioactive Scores

The bioactivity of a molecule is directly connected to the medicinal/pharmaceutical activity of the molecule. It is calculated in term of the bioactive scores, which are related to the binding preference of the molecule with the biological targets. The bioactive scores of the ME molecule are computed using the online software *Molinspiration* available at the site—www.molinspiration.com (accessed on 10 March 2023). The most common biological targets are the proteins like G protein-coupled receptor and nuclear receptor ligand, ion channel modulation, kinase inhibition, protease inhibition, and enzyme activity inhibition. The computed bioactive scores of the ME molecule are given in Table 3. These scores suggest that ME is a moderately bioactive molecule.

### 2.5. APT Charge

Atomic polar tensor (APT) charges at different sites of the three lower energy conformers are given in Table 4. The APT charges at various sites retain their polarity excepting the sites C_1_ and H_16_. The polarity of C_1_ and H_16_ atoms are found to be positive in conformers C-I and C-II and are found to be negative in the conformer C-III. The magnitudes of the APT charges are found to show variation in phenyl ring and allyl moieties, excepting the C atoms of the methyl groups. The C atoms of the phenyl ring attached to the substituents bear positive APT charges since all the three substituents are electron withdrawing in nature and the remaining C atoms of the phenyl ring bear negative APT charges. The magnitudes of the APT charges are found to be much higher at the C and O atoms of both the OCH_3_ groups as compared to the rest atoms. The highest negative charge is found to be at the O atom of the OCH_3_ group attached at the para- position relative to the allyl moiety. The H atoms attached to the C_15_ and C_17_ atoms have positive APT charges; however, the H atoms attached to the C_12_ atom bears negative APT charge in the allyl moiety. In going from one low energy conformer to another, a few sites show noticeable changes in the magnitudes of the APT charges. The enhancement in the magnitudes of the APT charges at the sites C_6_ and H_11_ in the conformer C-II and the site H_19_ in C-III is a result of steric hindrance with the allyl moiety. There is decrease in the APT charge at the site H_7_ in C-II and the sites C_2_ and C_5_ in the conformers C-II and C-III.

### 2.6. Vibrational Analysis

**Figure 3:**
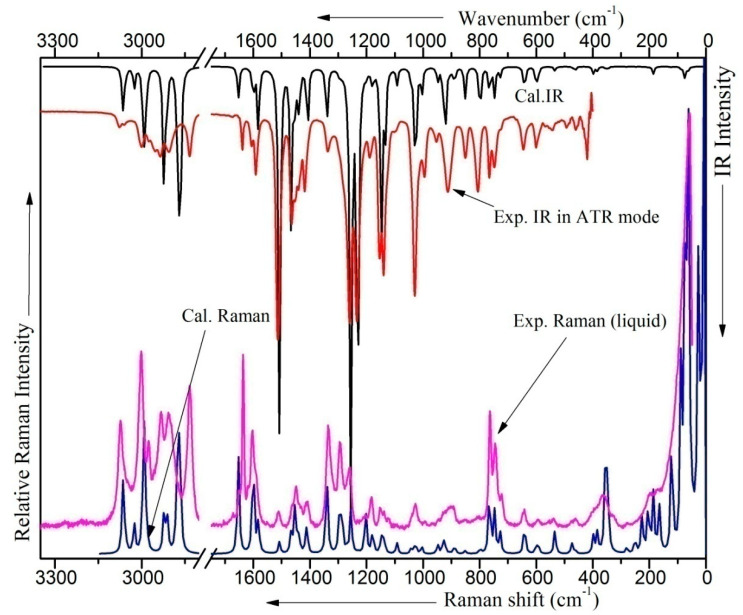
Computed (C-I) and observed IR and Raman of ME.

Table 5 presents the 75 normal modes of vibration of ME. The computed and experimental Raman and IR spectra are shown in Figure 3 and Figure S3. The observed IR and the Raman spectra of ME agree with the observed spectrum reported by the earlier authors [17]. The observed Raman and IR bands along with the corresponding computed scaled IR and Raman frequencies and their relative intensities, depolarization ratios of the Raman bands, PEDs, and the proposed mode assignments for the three lower energy conformers are collected in Table S3. Vibrational analysis has been made in light of the computed vibrational spectra and related quantities for the allyl and methoxy-benzenes. To correlate the experimental and computed scaled frequencies, help has also been taken from the vibrational assignments of the observed frequencies for the allyl-benzene (AB) [18], anisole [19], estragole (EG) [20], and eugenol (EU) [21] molecules. In order to correlate the normal modes of the phenyl ring moiety with benzene, the form of a normal mode, observed from the animation available with the Gauss View 05 software and the PEDs, was of considerable help.

The discussion of the vibrational assignments for the methyl eugenol molecule could be divided into three groups: (i) Methoxy (–OCH_3_) moiety modes (12 + 12), (ii) Allyl (–CH_2_–CH=CH_2_) moiety modes (21), and (iii) Phenyl moiety modes (30). 

#### 2.6.1. Methoxy (–OCH_3_) Group Modes (**24**)

The nine internal modes, namely υ_s_(a′), υ_as_(a″), υ_as_(a′), δ_as_(a′), δ_as_(a″), δ_s_(a′), ρ(a′), ρ (a″), and τ(CH_3_) of the CH_3_ groups attached to the C_3_ and C_4_ atoms, are computed to be 2867/2870, 2922/2924, 2993/2993, 1466/1467, 1454/1455, 1447/1438, 1175/1180, 1139/1139, and 225/250 cm^−1^, respectively. The magnitudes of the computed frequencies for the CH_3_ groups of ME agree within ±15 cm^−1^ with those of anisole and estragole [20] and eugenol [21], excepting the mode τ(CH_3_).

There is no observed frequency for the mode υ_s_(a′) in both the IR and Raman spectra of ME. For benzene derivatives with a methoxy group {anisole [19], EG [20], and EU [21]}, two frequencies are observed around 2835 and 2900 cm^−1^, which are explained to arise due to the Fermi resonance (FR) between the fundamental mode υ_s_(CH_3_) and the 1st overtone of the mode δ_s_(CH_3_). In the present case, the two frequencies 2834 and 2905/2907 cm^−1^ are observed in both the IR and Raman spectra. The computed (scaled) frequencies for the modes υ_s_(CH_3_) and δ_s_(CH_3_) are found to be 2867/2870 and 1447/1438 cm^−1^. Therefore, the average (2870 cm^−1^) of the two observed frequencies—2834 and 2905 cm^−1^—is assigned to the mode υ_s_(CH_3_) and the two frequencies 2834 and 2905 cm^−1^ are explained to arise due to the FR between υ_s_(CH_3_) and 2 × δ_s_(CH_3_). The δ_s_(CH_3_) modes for both OCH_3_ groups are observed at 1441(IR)/1449(R) cm^−1^.

The modes υ_as_(CH_3_)(a″) and υ_as_(CH_3_)(a′) were correlated with the observed frequencies 2905(IR)/2908(R) and 3001(IR)/3002(R) cm^−1^ and the a′ and a″ components of the anti-symmetric deformation modes are observed at 1464(IR/R) and 1452(IR) cm^−1^, respectively. The CH_3_ torsional modes corresponding to the OCH_3_ groups attached to the meta- and para- C atoms relative to the C atom to which the allyl group is attached are computed to be 225 and 250 cm^−1^. In estragole, the OCH_3_ group is attached to the para- C atom, while in eugenol it is attached to the meta- C atom relative to the allyl attached C atom of the ring. Moreover, the τ(CH_3_) mode is found to have magnitudes 223 and 246 cm^−1^ for the eugenol and the estragole molecule. Therefore, the magnitudes of the τ(CH_3_) modes of ME also agree with those of eugenol and estragole considering the position of the C atom of the ring to which the OCH_3_ group is attached relative to the allyl C atom of the ring.

The modes υ_s_(a′), υ_as_(a″), δ_as_(a″), ρ(a″), and τ(CH_3_) of one OCH_3_ group are found to be coupled with the corresponding modes of the other OCH_3_ group. Moreover, the δ_s_(CH_3_) mode of both the CH_3_ groups appear to be coupled with the β_s_(–CH_2_) mode of the allyl moiety. The frequencies corresponding to the modes υ_s_(a′), υ_as_(a″), υ_as_(a′), δ_s_(a′), and ρ(a″) of the ^9^OCH_3_ group are computed to be lower than the corresponding modes of the ^8^OCH_3_ group.

In light of the PEDs of the scaled frequencies, 1028/1041, 593/384, and 89/65 cm^−1^ correspond to the modes ν(O–CH_3_), α(C–O–CH_3_), and τ(C–OCH_3_) for the ^9^OCH_3_/^8^OCH_3_ groups. The frequency 1028 cm^−1^ involves the modes ν(O_9_–C_20_) and ν(O_8_–C_24_) with contributions 43% and 16%, whereas the frequency 1041 cm^−1^ involves the modes ν(O_8_–C_24_) and ν(O_9_–C_20_) with contributions 47% and 27%. Further, the frequency 1028 cm^−1^ has major contribution from the mode ν(O_9_–C_20_) having out-of-phase coupling (opc) with the ν(O_8_–C_24_) mode. Similarly, the frequency 1041 cm^−1^ has major contribution from the mode ν(O_8_–C_24_) having in-phase coupling (ipc) with the ν(O_9_–C_20_) mode. The frequencies 1028 and 1041 cm^−1^ are correlated with the observed bands 1029(IR)/1027(R) cm^−1^ and 1039(R) cm^−1^, respectively. Likewise, the modes α(C–O–CH_3_) and τ(C–OCH_3_) of one OCH_3_ group are also found to couple with the corresponding modes of the other OCH_3_ group as well as some other modes of the allyl and phenyl moieties. The mode α(C–O–CH_3_) is observed at 538(IR)/382(R) cm^−1^ with weak intensity in the Raman spectrum. The mode τ(C–^9^OCH_3_) is observed as a strong Raman band of 60 cm^−1^. The assignment of the modes ν(O–CH_3_) and τ(C–OCH_3_) are in support with assignment of the modes in the anisole [19], the estragole [20], and the eugenol [21] molecules. The mode α(C–O–CH_3_) shows variation in going from one molecule to another as well as surrounding of the substituent groups.

Out of the 12 modes of each OCH_3_ group, the 3 modes α(C–O–CH_3_), τ(C–OCH_3_), and τ(CH_3_) show a noticeable change in computed frequencies in going from one low energy conformer to another. The mode τ(CH_3_) of the CH_3_ group attached to the meta- C atom relative to the C atom attached with allyl group is reduced by 17 cm^−1^ in the conformer C-III relative to the conformers C-I and C-II.

#### 2.6.2. Allyl (–CH_2_–CH=CH_2_) Group Modes (**21**)

##### Methylene (–CH_2_–) Group Modes (**6**)

The modes ν_s_ and ν_as_ were found to be pure modes with the scaled frequencies 2879 and 2911 cm^−1^, respectively. The mode β_s_ (1439 cm^−1^) has strong coupling with the mode δ_s_(CH_3_) of the ^8^OCH_3_ group. Similar to the case of the OCH_3_ group(s), the ν_s_ and 2× β_s_ of the methylene group are found to be in FR with each other, giving rise to the two observed component frequencies as 2905(IR)/2907(R) and 2834(IR, R) cm^−1^. The average of these two frequencies, i.e., 2870 cm^−1^ is assigned to the mode ν_s_and the observed frequency 1444 cm^−1^ to the mode β_s_ of the methylene group. It is noteworthy that both the modes ν_s_(–CH_3_) and ν_s_(–CH_2_) are correlated to the same pair of the observed frequencies 2906 and 2834 cm^−1^. Likewise, the modes δ_s_(–CH_3_) and β_s_(–CH_2_) are correlated to the same observed frequencies 1445(IR)/1449(R) cm^−1^. The observed frequency corresponding to the mode ν_as_(–CH_2_–) is computed to be 2911 cm^−1^ and appears to be obscured with the higher frequency component (2906 cm^−1^) of the FR doublet.

The modes ω, ρ, and t of methylene group were computed to be 1297, 1203, and 889 cm^−1^, respectively, and appear to be strongly coupled with the other modes (Table S3). The t mode is not a usual torsional mode, which makes its magnitude considerably high (889 cm^−1^) compared to the usual torsion modes (<500 cm^−1^). For the EU [21], it is observed at 894 cm^−1^ in the Raman spectrum. The modes ω and ρ are observed in the Raman spectrum at the frequencies 1293 and 1206 cm^−1^. The assignments of these modes are in agreement with the corresponding modes of the EG [20] and EU [21] molecules.

##### Vinyl (–CH=CH_2_) Group Modes (**12**)

The three C–H stretching modes ν_as_(=CH_2_), ν(=C–H), and ν_s_(=CH_2_) of the vinyl (–CH=CH_2_) group are computed to be 3068, 2991, and 2984 cm^−1^, respectively. The highest frequency 3068 cm^−1^ mode is a result of the ν_as_(=CH_2_) mode with 98% contribution. The frequency 2991 cm^−1^ arises due to the ipc between the modes ν(=C–H) and ν_s_(=CH_2_) with the contributions 71% and 27%, whereas the frequency 2984 cm^−1^ arises due to the opc between the above two modes with the contributions 26% and 73%, respectively. The observed frequencies 3077(IR)/3073(R), 3001(IR)/3002(R), and 2976(IR, R) cm^−1^ are correlated to the modes ν_as_(=CH_2_), ν(=C–H) and ν_s_(=CH_2_), respectively.

The three C–H planar bending modes β_s_(=CH_2_), δ(=C–H), and ρ(=CH_2_) of the vinyl group are correlated to the computed frequencies 1414, 1287, and 1092 cm^−1^, respectively. The modes β_s_(=CH_2_) and ρ(=CH_2_) are found to be strongly coupled with the other modes of the allyl moiety; however, the δ(=C–H) mode has strong coupling with the ring β(C–H) modes and weak coupling with other allyl moiety modes. The observed frequencies corresponding to these modes are identified as 1418(IR)/1412 (R), 1285 (IR), and 1101 (IR) cm^−1^, respectively.

The computed frequencies 1005, 920, and 600 cm^−1^ arise due to non-planar bending motions of the three C–H bonds of the vinyl moiety. The frequency 1004 cm^−1^ is found to arise due to the opc of the τ(=CH_2_) mode with the γ(=C–H) mode, whereas the ipc between these two modes yields the frequency 600 cm^−1^. The remaining ω(=CH_2_) mode has a strong coupling with the ν(C_12_–C_15_) mode. These frequencies are correlated to the observed frequencies 995(IR)/994(R), 913(IR)/923(R), and 601(IR)/596(R) cm^−1^, respectively. The present assignments for the allyl group modes are in agreement with the corresponding assignments reported earlier [18,20,21].

The three modes ν(C=C), α(C_12_–C_15_=C_17_), and τ(C_12_–C_15_) are computed to be 1650, 279, and 75 cm^−1^, respectively. The modes α(C_12_–C_15_=C_17_) and τ(C_12_–C_15_) are found to be strongly coupled with the other modes. The vibrational frequencies show the variation in going from one low energy conformer to another for a few modes of the allyl moiety (Table S3).

#### 2.6.3. Phenyl Moiety Modes (**30**)

The phenyl ring moiety consists of three parts: (i) phenyl ring, (ii) C-H bonds, and (iii) 2 C–O(CH_3_) and C–C(H_2_CHCH_2_) bonds. The assignments for these three parts are discussed separately in the following three sub-sections.

##### Phenyl Ring Modes (**12**)

The magnitudes of the ring stretching modes 8a, 8b, 19a, and 19b are found to be similar to those for the estragole, eugenol, and ME molecules. For the ME molecule, these modes were computed to be 1600, 1581, 1507, and 1407 cm^−1^ with the respective observed frequencies 1605(IR, w)/1604(R, s), 1591(IR, m)/1591(R, m), 1514(IR, s)/1511(R, m), and 1418(IR, m)/1412(R, w) cm^−1^. Kekule ring stretching mode 14 shows variation and is found to have frequencies of 1325, 1367, and 1340 cm^−1^ for the estragole, eugenol, and ME molecules, respectively. The ring breathing mode 1 is computed to be 765 cm^−1^ with the observed frequency 766 cm^−1^ in both the IR and Raman spectra with good intensities. The present assignments are in agreement with the corresponding assignment made by Chowdhry et al. [17] for the ME molecule.

For the ME molecule, the modes 4, 16a, and 16b are computed to be 728, 748, and 463 cm^−1^, respectively, with the observed frequencies of 724(IR)/722(R), 748(IR)/745(R), and 460(IR/461(R) cm^−1^. The assignments for the modes 4 and 16b are in agreement with eugenol; however, the mode 16a is found to have a higher frequency (748 cm^−1^) compared to eugenol (597 cm^−1^). The modes 6a, 6b, and 12 are computed to have frequencies of 473, 534, and 642 cm^−1^, respectively, with the corresponding observed frequencies of 483(IR)/473(R), 542(IR)/547(R), and 646(IR)/647(R) cm^−1^. Mode 12 is found to involve two frequencies: 1028 and 642 cm^−1^. The higher frequency also involves the O–CH_3_ stretching of both the methoxy groups and is more suitable for the opc O-CH_3_ stretching mode. Therefore, the lower frequency is assigned to mode 12 of ME. Mode 12 is found to be lower (645 cm^−1^) in ME as compared (743 cm^−1^) to the eugenol molecule. For the other two modes, the present assignments agree with those of the eugenol molecule.

##### C–H Modes (**9**)

The computed stretching frequencies for the three C–H bonds C_5_–H_10_, C_2_–H_7_, and C_6_–H_11_ are 3063, 3056, and 3026 cm^−1^, respectively, with the observed frequencies of 3061(IR), 3049(R), and 3037(R) cm^−1^, respectively. The highest frequency β(C–H) mode is computed to be 1269 cm^−1^ and is not observed in both the IR and Raman spectra and corresponds to the mode 3 of benzene. The computed frequencies 1149 and 1134 cm^−1^ result due to the two β(C–H) modes assigned to the observed frequencies 1153(IR)/1152(R) and 1122(R) cm^−1^, respectively. Both these frequencies are found to couple with other modes. The frequency 1149 cm^−1^ is found to couple strongly with the mode ν(C_1_–C_12_) and weakly with the modes ν(C–O) and ν(O–CH_3_) of both the methoxy groups.

The computed frequencies for the three γ(C–H) modes are 903, 851, and 800 cm^−1^, the latter two being observed at 850(IR)/853(R) and 806(IR)/805(R) cm^−1^ having weak Raman and medium IR intensities. The highest/lowest γ(C–H) mode arises due to the opc/ipc of the C–H bending motions at the positions C_5_ and C_6_ with a small contribution from the ring deformation modes. The remaining γ(C–H) mode arises due to non-planar bending of the C_3_–H bond. The present assignments of the modes ν(C–H), β(C–H), and γ(C–H) are supported by the assignments proposed by Chowdhry et al. [17] for ME.

##### C–O(CH_3_) and C–C(H_2_CHCH_2_) Group Modes (**9**)

The two ν(C–O(CH_3_)) modes are computed to be 1231 and 1255 cm^−1^. These modes are coupled with each other as well as with the ring modes. The frequencies 1231 and 1255 cm^−1^ arise due to the opc and ipc of the stretching motions of the C–OCH_3_ bonds of both the methoxy groups and are observed at 1235(R) and 1260(IR)/1259(R) cm^−1^, respectively. The frequency 1231 cm^−1^ arises due to the ipc of the modes ν(C_3_–O(CH_3_)) and ν(C_4_–O(CH_3_)) with the contributions 9% and 24%, respectively, whereas the frequency 1255 cm^−1^ arises due to the opc of the same modes with the contributions 16% and 11%, respectively. The assignments for these modes are in agreement with the assignments reported by Chowdhry et al. [17] for ME. The ν(C_1_–C_12_) mode is calculated to be 928 cm^−1^ and could not observed in both the IR and Raman spectra of ME. The assignment of this mode is in agreement with the assignment for this mode in estragole [20]. The β(C_1_–C_12_) and γ(C_1_–C_12_) modes are computed to be 345 and 122 cm^−1^, respectively, and lie outside the investigated IR range and could not be observed in Raman spectrum. The planar and non-planar bending modes for both the C–O(CH_3_) groups attached to the sites C_3_ and C_4_ are computed to be 354/185 and 204/166 cm^−1^ with the corresponding observed frequencies of 361/196 and -/170 cm^−1^.

For the C–C(H_2_CHCH_2_) bond, the ν(C–C(H_2_CHCH_2_)) mode is computed to be 939 cm^−1^. From the PEDs, this mode is found to couple strongly with the modes of the allyl moiety and trigonal ring deformation mode 12. The planar and non-planar bending modes are computed to be 291 and 174 cm^−1^ and observed at 300 and 179 cm^−1^, respectively, in the Raman spectrum. The assignments of these modes are in agreement with the assignments of the corresponding modes for the estragole molecule [20].

### 2.7. Conformer Dependent Modes

The computed IR and Raman spectra for the three lower energy conformers are shown in Figure S4. The corresponding computed frequencies for the conformers C-I and C-II are very close to each other. However, the conformer C-III has 15 fundamental modes with computed frequencies having difference greater than 10 cm^−1^ as compared with the corresponding modes of the conformers C-I/C-II. Only two of these modes are observed separately, while the remaining 13 cannot be observed for C-III. These two modes are the α(C_14_–C_15_=C_17_) and α(R)-6a, which could be correlated to the observed frequencies 420 and 553 cm^−1^, respectively, for the conformer C-III. These two modes were observed at 403 and 542(IR)/538(R) cm^−1^ for the conformers C-I/C-II. The frequencies 420 and 553 cm^−1^ correlated to the C-III modes could also be interpreted as a combination (165 + 250 = 415 cm^−1^) and overtone (2 * 279 cm^−1^) bands of the conformer C-I. Thus, the entire experimental IR and Raman spectra could be explained in terms of the computed IR and Raman spectra of the lowest energy conformer C-I.

### 2.8. Solvent Effects

**Figure 4:**
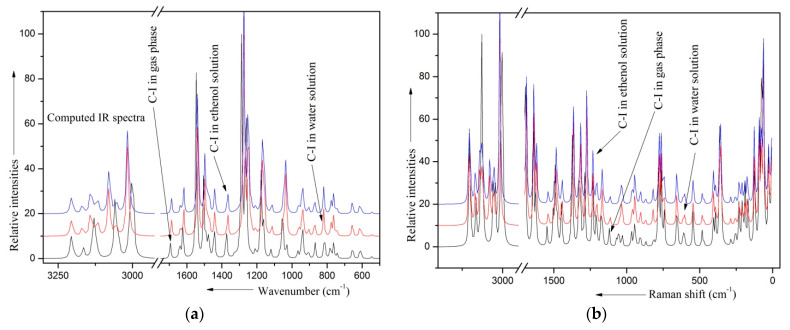
Computed (**a**) IR and (**b**) Raman spectra of C-I monomer in gas phase and solvent medium.

**Figure 5:**
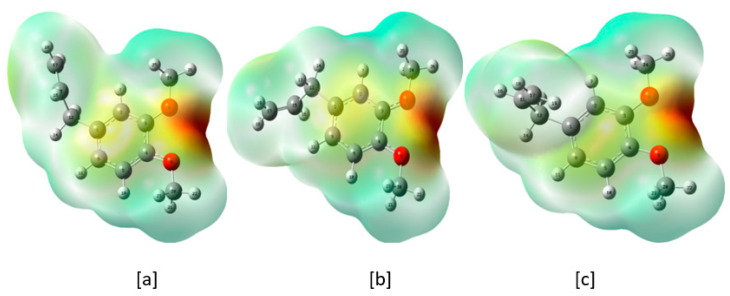
(**a**–**c**) MEP plots of the three lower energy conformers of ME.

The two solvents water and ethanol are found to have almost same influence on different molecular properties. In the presence of water and ethanol as solvents, the geometrical parameters do not change as compared to their gas phase structure, excepting the parameters related to the two C–OCH_3_ moieties. For each OCH_3_ group, the bond lengths C–O and O–CH_3_ were found to be increased by 0.003 and 0.008 Å, respectively, relative to the gas phase values. The angles of the CH_3_ moieties are found to show variations in the range 0.2–0.4°. The magnitudes of APT charges at different sites are found to enhance significantly in several cases, the largest enhancement being at the sites of the twp O atoms. The sites of the H- atoms are found to show minimum enhancement. The computed IR and Raman spectra for the lowest energy conformer C-I in gas phase and solvent medium are shown in Figure 4. The computed vibrational frequencies and related parameters in gas and solvent medium are listed in Table 6. This table shows that the major of the modes show a change in frequencies. Out of 75 modes, 3 modes—τ(C_1_–C_12_), α_3_(R)-6b, and Φ(R)-16a—do not show variation in frequency; of the remaining 72 modes, only 22 modes show considerable change in frequencies. For 22 modes, the computed frequencies are found to show variation in the range 6–20 cm^−1^ in the presence of the solvents. Out of these modes, 14 modes—γ(C–H)-17a, ρ(=CH_2_), β(C–H), ν(R)-14, β_s_(=CH_2_), δ_as_(CH_3_)-a″, δ_as_(CH_3_′)-a″, δ_as_(CH_3_)-a′, δ_as_(CH_3_′)-a′, ν(R)-19a, ν(C=C), ν_as_(–CH_2_), ν_as_(CH_3_)(a′), and ν_as_(CH_3_′)(a′)—have frequency differences in the range 6–10 cm^−1^, while 8 modes—ν(C–O)_ipc_, ν(C–O)_opc_, ν(^8^O–CH_3_), ν(^9^O–CH_3_), ν_s_(CH_3_′), ν_s_(CH_3_), ν_as_(CH_3_′)(a′), and ν_as_(CH_3_)(a′)—have frequency differences in the range 10–20 cm^−1^. All these modes are associated with either the ring or the OCH_3_ groups modes. The variations in the geometrical parameters and vibrational frequencies related to the two methoxy moieties suggest that the solvent–solute interaction is effective near the electron-rich site (red color region in the MEP plots (Figure 5)). The depolarization ratios of the Raman bands also show considerable variation for 17 modes, out of which 10 modes—ν(R)-19a, ν(R)-19b, δas(CH_3_)-a′, ν(C–O)_opc_, ρ(CH_3_′)-a′, ρ(CH_3_)-a″, β(C–H)-8a, ν(^9^O–CH_3_), β(C_3_–O_8_), and ν(C_12_–C_15_)—have reduced depolarization ratios, while 7 modes—δ_as_(CH_3_′)-a′, δ_s_(CH_3_′)-a′, ω(=CH_2_), γ(C–H), α(C–O–C)_ipc_, α(C–O–C)_opc_, and β(C_1_–C_12_)—have enhanced depolarization ratios. The enhanced/reduced depolarization ratios arise due to less/more symmetric nature of the corresponding mode. The Raman intensities are found to show considerable variations for 14 modes, out of which intensities are reduced for 12 modes, namely, ν(C–H)-2, ν_as_(CH_3_)(a′), ν_as_(CH_3_′)(a′), ν_s_(=CH_2_), ν_as_(CH_3_)(a″), ν_s_(CH_3_)(a′), ν_s_(CH_3_′)(a′), ν(C=C), ω(–CH_2_), β(C–H)-8a, β(C_3_–O_8_), and γ(C_3_–O_8_), while the modes ν_s_(CH_2_) and ν(C–O)_opc_ are found to show enhanced Raman intensities. The IR intensities are found to show little variation for all the modes, excepting β(C–H)-8a, ν(C–O)_opc_, ν_as_(CH_3_′)(a′), ν(R)-19a, and ν_s_(CH_3_)(a′), for which the IR intensities are reduced. This suggests that the presence of solvents during these modes of vibration affect the permanent as well as the induced dipole moments considerably.

### 2.9. MEP Plots

Molecular electrostatic potential (MEP) plot is helpful in locating the active sites near the molecule [22,23,24]. The MEP plots for the first three lower energy conformers are depicted in Figure 5. The blue color represents the highest positive charge density and the red color represents the highest negative charge density. The blue color is the most suitable site for the nucleophilic substitution and the red color is the most suitable site for the electrophilic substitution. For the ME molecule, the red color is spread between the O atoms of both methoxy moieties. The light green color is spread near the H atoms of both methoxy groups. Thus, the strong electrophilic substitution would take place in the proximity of the O atoms of the OCH_3_ groups. The proximity of the H atoms of the methoxy moieties is suitable for a weak nucleophilic substitution (Figure 5).

### 2.10. HOMO-LUMO Plots

**Figure 6:**
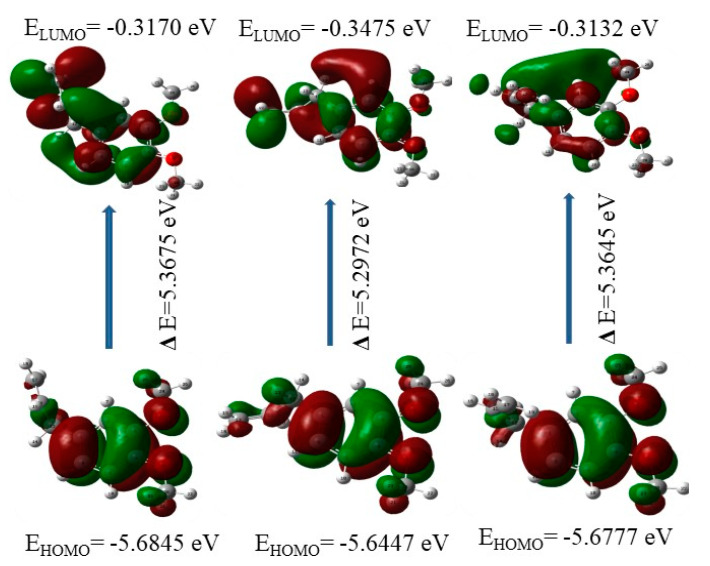
HOMO, LUMO with their energies and gaps in the three lower energy conformers.

Energies of HOMO (E_H_) and LUMO (E_L_) are used to estimate the chemical parameters like chemical softness, ionization potential, electron affinity, and electrophilicity index, etc. The energies (-E_H_), (-E_L_) and their gap (E_H_–E_L_) represent the ionization potential, electron affinity, and chemical hardness, respectively [25,26,27,28,29]. The magnitude of the energy gap (E_H_–E_L_) gives the reactive behavior of the molecule. The HOMO-LUMO plot of the ME molecule is given in Figure 6. The E_H_, E_L_, and E_H_–E_L_ and the calculated parameters from these are given in Table 7. The value of E_H_–E_L_ suggests that the molecule is chemically soft in nature. From the HOMO plot, it could be seen that the electron density is delocalized mainly over the phenyl ring and O atoms of the OCH_3_ groups. However, in the LUMO, the charge density is shifted from the methoxy moieties towards the allyl moiety.

**Figure 7:**
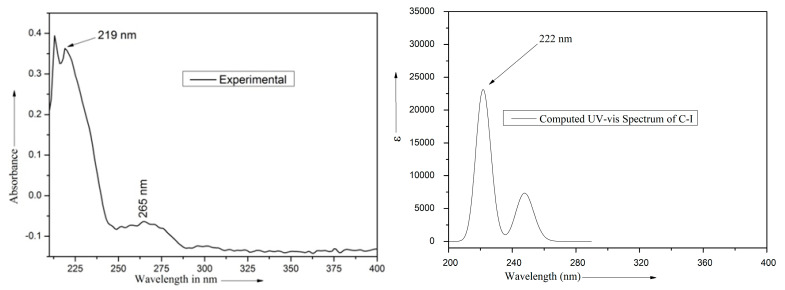
Observed and computed UV-vis spectra of ME.

The computed and observed UV-vis spectra of ME are shown in Figure 7. The computed peak positions, related quantities, the possible transitions for the computed peaks and the observed peaks are listed in Table 8. The contribution of the HOMO → LUMO + 3 transition to the computed peak 248 nm is 45%. This computed peak could be correlated with the observed peak 265 nm. The remaining two computed peaks 223 and 220 nm have major contributions from the transitions HOMO → LUMO + 1 and HOMO → LUMO + 10 and appear to merge into a single band with peak at 222 nm which correspond to the observed band with peak at 219 nm.

## 3. Experimental Details

The methyl-eugenol compound is found as a colorless liquid, purchased from the Sigma Chemical Co., (St. Luis, MO, USA) and used as such for the spectral study. The IR spectrum was recorded in ATR mode on a Perkin Elmer spectrometer rx-1 within the range 400–4000 cm^−1^_,_ using the following parameters: scan −10, gain −50, and resolution −2 cm^−1^. The Raman spectrum was recorded on a Renishaw in Via Raman microscope in the liquid form in the region 50–4000 cm^−1^ equipped with a 2400 line/mm grating. The sample was mounted in the sample illuminator using an optical mount without any kind of pre-treatment. The diode laser of wavelength 532 nm was used to excite the spectrum. The laser power was set at 50% of 50 mW and at 50× objective lens, acquisition time—10 s, and spectral resolution—1.0 cm^−1^. The UV-vis spectrum was recorded in absorption mode in the range 200–600 nm in ethanol with ~10^−4^ mole/L concentration on an Eppendorf Bio-spectrometer, model kinetic (Hellma GmbH & Co.KG, Müllheim, Germany).

## 4. Computational Detail

All the computations were carried out using the Gaussian 09 program [30] at the level B3LYP/6-311++G**. The geometries of ME and its conformers were optimized by minimizing the energies with respect to all the geometrical parameters without imposing any molecular symmetry constraints. To determine the total number of possible conformers, we scanned the total energy versus the dihedral angle surface for different tops. The estimated Raman activities (S_i_) from the Gaussian 09 software were converted into the Raman intensities (I_i_) using the relation given elsewhere [31]. The PEDs for all the normal modes of vibration were computed using the GAR2PED software (1996) [32]. Molecular electrostatic potential (MEP) and HOMO and LUMO energies were calculated at the same level. The UV-vis spectrum was calculated using the TD-DFT CAM-B3LYP method with the basis set 6-311++G**. The bioactivity scores of the ME molecule were computed using the Molinspiration online software (1987).

## 5. Conclusions

The ME molecule is found to possess 21 stable conformers. Corresponding to each conformer, there are two molecules of the same energy which are enantiomers of each other. Two conformers (C-II and C-III) have energies below the room temperature with respect to the lowest energy conformer C-I. In going from one low energy conformer to another, the geometrical parameters do not show considerable variation, except the C_12_–C_15_ bond, which is enhanced in C-II by 0.006 Å as compared to C-I/C-III. The barrier heights suggest that the transition C-II → C-I is more probable compared to the reverse transition. The assignments of the vibrational frequencies of ME are found to agree well with the corresponding modes of the related molecules, like allyl-benzene, anisole, estragole, and eugenol. All the modes of both CH_3_ groups seem to be pure modes, except the τ(CH_3_) modes. The modes of one C–O–C group are strongly coupled with the corresponding modes of the other C–O–C group. Out of the 21 allyl group modes, the 8 higher frequency modes are found to be pure modes, whereas the remaining 13 modes are coupled with the phenyl ring modes and other modes of the same moiety. Similar to the case of estragole and eugenol, the ME molecule also has the same observed Fermi doublets (2835 and 2905 cm^−1^) for the two pairs of resonating modes—ν_s_(–CH_2_) and 2 × β_s_(–CH_2_); ν_s_(CH_3_) and 2 × δ_s_(CH_3_). The presence of solvent is seen to significantly affect the geometrical parameters related to the OCH_3_ groups. However, the magnitudes of the APT charges at different sites are found to enhance considerably. The solute–solvent interaction results in noticeable changes in the vibrational frequencies and related parameters.

From the MEP plots, the most electron-rich site is seen to exist in the space between both the O atoms of the methoxy groups and would be a suitable site for the electrophilic substitution to take place. The most electron-deficient sites are in the proximity of the H atoms of both the CH_3_ groups and are suitable for nucleophilic substitutions. The HOMO-LUMO analysis also suggested that the ME molecule is chemically softer compared to its family members like estragole and eugenol. The bioactive scores lie in the range 0–(−5), suggesting that ME is moderately bioactive in nature.

## Figures and Tables

**Table 1:** Total and relative energies of all the 21 conformers of ME.

Conformers	Energy (E)	ΔE Relative to the Conformer I		Relative Abundance
	kcal/Mole	kcal/Mole	K	
I	−362,794.265	0	0	32.1
II	−362,793.860	0.405	204	16.2
III	−362,793.644	0.622	313	11.2
IV	−362,793.237	1.028	518	5.7
V	−362,793.212	1.054	530	5.4
VI	−362,792.997	1.268	638	3.8
VII	−362,792.978	1.287	648	3.7
VIII	−362,792.903	1.363	685	3.2
IX	−362,792.881	1.384	697	3.1
X	−362,792.871	1.395	701	3.1
XI	−362,792.859	1.408	708	3.0
XII	−362,792.61	1.655	833	2.0
XIII	−362,792.593	1.672	842	1.9
XIV	−362,792.517	1.748	880	1.7
XV	−362,792.517	1.749	880	1.7
XVI	−362,791.789	2.477	1246	0.5
XVII	−362,791.74	2.525	1271	0.5
XVIII	−362,791.725	2.541	1279	0.4
XIX	−362,791.705	2.561	1289	0.4
XX	−362,791.302	2.963	1491	0.2
XXI	−362,791.301	2.964	1492	0.2

**Table 2:** Barrier heights for all the tops of ME.

Top	Foldness	* ∆E_AB_	* ∆E_BC_	* ∆E_CD_	* ∆E_DE_	* ∆E_EF_	* ∆E_FG_
CHCH_2_	3	2.36	1.27	2.00	1.98	3.62	4.73
CH_2_CHCH_2_	2	2.02	1.60	1.52	1.94	-	-
CH_3_	3	3.34	3.34	3.34	3.34	3.34	3.34
CH_3_	3	3.34	3.34	3.34	3.34	3.34	3.34
^8^OCH_3_	3	1.13	1.54	7.15	7.15	1.54	1.13
^9^OCH_3_	3	2.37	1.18	2.73	2.71	1.15	2.36
=CH_2_	2	93.13	93.13	93.13	93.13	-	-

* Barrier heights in kcal/mole.

**Table 3:** Bioactive Scores of ME.

Bioactive Acceptors	M-Eugenol
GPCR ligand	−0.81
Ion channel modulator	−0.38
Kinase inhibitor	−1.06
Nuclear receptor ligand	−0.80
Protease inhibitor	−1.14
Enzyme inhibitor	−0.43

**Table 4:** APT charges (in e unit) at different atomic sites of ME.

Atoms	C-I	C-II	C-III	C-I In Solvent Effect	
				Water	Ethanol
C_1_	0.018	0.012	−0.004	0.040	0.039
C_2_	−0.159	−0.119	−0.117	−0.200	−0.198
C_3_	0.515	0.516	0.503	0.688	0.680
C_4_	0.518	0.510	0.513	0.668	0.661
C_5_	−0.096	−0.072	−0.088	−0.131	−0.129
C_6_	−0.086	−0.120	−0.080	−0.114	−0.112
H_7_	0.062	0.050	0.050	0.087	0.085
O_8_	−0.863	−0.891	−0.869	−1.135	−1.121
O_9_	−0.922	−0.919	−0.907	−1.173	−1.161
H_10_	0.045	0.045	0.046	0.075	0.073
H_11_	0.027	0.042	0.027	0.040	0.039
C_12_	0.133	0.131	0.133	0.154	0.153
H_13_	−0.051	−0.053	−0.041	−0.058	−0.058
H_14_	−0.028	−0.032	−0.035	−0.039	−0.038
C_15_	0.048	0.061	0.064	0.084	0.082
H_16_	0.018	0.016	−0.021	0.018	0.018
C_17_	−0.141	−0.142	−0.149	−0.200	−0.196
H_18_	0.029	0.028	0.023	0.039	0.038
H_19_	0.029	0.029	0.046	0.038	0.038
C_20_	0.552	0.542	0.545	0.653	0.648
H_21_	−0.043	−0.040	−0.041	−0.043	−0.043
H_22_	−0.002	−0.002	−0.001	−0.003	−0.003
H_23_	−0.042	−0.041	−0.041	−0.042	−0.042
C_24_	0.511	0.529	0.520	0.631	0.625
H_25_	−0.035	−0.039	−0.038	−0.039	−0.039
H_26_	0.000	−0.001	0.000	0.000	0.000
H_27_	−0.038	−0.040	−0.039	−0.040	−0.039

**Table 5:** Normal mode distribution of ME.

Group/Moiety		Modes in Symbolic Form *	Total
Phenyl	Ring	6ν(R) + 3α(R) + 3Φ(R)	12
	(Ph)C–O(CH_3_)	2ν + 2β + 2γ	6
	(Ph)C–C(H_2_CHCH_2_)	ν + β + γ	3
	(Ph)C–H	3ν + 3β + 3γ	9
OCH_3_	CH_3_	2ν_s_ + 4ν_as_ + 2δ_s_ + 4δ_as_ + 2ρ_║_ + 2ρ_┴_ + 2τ	18
	O–C(H_3_)	2ν + 2α + 2τ	6
Allyl	2CH_2_	2ν_s_ + 2ν_as_ + 2β_s_ + 2ρ + 2ω + t + τ	12
	C–C	ν + α + τ	3
	C–H	ν + 2δ	3
	C=C	ν + α + τ	3

* α(R)—is planar ring deformation, υ(R)—is ring stretching, Φ(R)—is non planar ring deformation, β—planar bending, γ—non planar bending, β_s_—is scissoring of group, ρ—is rocking of group, ω—is wigging of the group, τ—is torson of group, t—is twisting of group, υ_as_—is Anti-symmetric stretching, υ_s_—is symmetric stretching, δ—angle deformation of group δ_s_, δ_as_—are symmetric and anti-symmetric angle deformations of the CH_3_ group.

**Table 6:** Solvent effect on **^#^** vibrational frequencies of the lowest energy conformer (C-I) of ME.

Gas	Solvent				# Mode
	Water	Ethanol			
(1)	(2)	(3)	(1)–(2)	(1)–(3)	
28 (0.29) 0.74	27 (0.16) 0.75	27 (0.21) 0.75	1	1	τ(C_1_–C_12_)
64 (1.95) 0.69	61 (1.53) 0.75	62 (1.63) 0.75	3	2	τ(C_4_–OCH_3_)
76 (2.48) 0.71	77 (2.24) 0.75	78 (2.29) 0.75	−1	−2	τ(C_12_–C_15_)
89 (0.40) 0.74	91 (1.31) 0.75	91 (1.39) 0.75	−2	−2	τ(C_3_–OCH_3_)
121 (0.36) 0.75	122 (0.25) 0.75	123 (0.31) 0.75	−1	−2	γ(C_1_–C_12_)
165 (0.17) 0.73	169 (0.11) 0.73	169 (0.13) 0.73	−4	−4	γ(C_3_–O_8_)
186 (2.23) 0.50	187 (2.9) 0.37	187 (2.11) 0.37	−2	−2	β(C_3_–O_8_)
204 (0.21) 0.62	207 (0.6) 0.68	207 (0.8) 0.68	−3	−3	γ(C_4_–O_9_)
225 (0.18) 0.69	224 (0.9) 0.75	224 (0.12) 0.75	1	1	τ(OCH_3_)
250 (0.7) 0.68	251 (0.3) 0.67	251 (0.4) 0.66	−1	−1	τ(OCH_3_)
279 (0.3) 0.33	282 (0.2) 0.60	282 (0.3) 0.60	−3	−3	α(C_1_–C_12_–C_15_)
345 (0.18) 0.25	344 (0.5) 0.58	344 (0.7) 0.55	2	2	β(C_1_–C_12_)
354 (0.74) 0.11	353 (0.44) 0.10	353 (0.49) 0.10	1	1	β(C_1_–O_10_)
384 (1.12) 0.22	383 (1.5) 0.43	383 (1.7) 0.42	1	1	α(C–O–C)ipc
398 (2.10) 0.72	397 (1.12) 0.73	397 (1.14) 0.73	1	1	α(C_14_–C_15_=C_17_)
462 (1.2) 0.69	462 (1.2) 0.68	462 (1.2) 0.69	1	1	Φ(R) 16b
474 (0.6) 0.61	473 (0.3) 0.71	473 (0.4) 0.71	0	0	α(R) 6b
534 (1.15) 0.52	533 (1.11) 0.46	533 (1.13) 0.47	1	1	α(R) 6a
592 (1.5) 0.54	590 (1.3) 0.51	591 (1.4) 0.52	3	2	α(C–O–C)opc
600 (4.6) 0.72	601 (5.3) 0.71	601 (5.4) 0.71	−1	−1	τ(C=C)
642 (6.22) 0.49	643 (7.15) 0.46	643 (6.19) 0.46	−1	−1	α(R) 12
728 (2.15) 0.17	730 (3.15) 0.25	730 (3.18) 0.24	−2	−2	Φ(R) 4
748 (7.30) 0.13	748 (9.25) 0.14	748 (9.31) 0.14	0	0	Φ(R) 16a
766 (6.47) 0.07	762 (6.27) 0.07	763 (6.34) 0.07	3	3	ν(R) 1
799 (12.3) 0.24	803 (12.6) 0.57	803 (12.7) 0.56	−5	−4	γ(C–H)
851 (7.2) 0.13	854 (8.3) 0.20	854 (8.3) 0.19	−2	−2	γ(C_2_–H_7_)
888 (4.6) 0.14	887 (4.7) 0.13	888 (4.8) 0.13	2	1	t(C_12_–H)
903 (1.1) 0.44	912 (2.0) 0.41	911 (2.0) 0.48	−9	−9	γ(C–H)
920 (12.3) 0.47	919 (10.5) 0.67	919 (10.5) 0.65	1	1	ω(=CH_2_)
928 (6.11) 0.48	927 (5.10) 0.67	927 (6.13) 0.66	2	1	ν(C_12_–C_15_)
948 (3.7) 0.05	943 (5.9) 0.05	943 (5.10) 0.05	5	5	ν(C_1_–C_12_) 20a
1005 (6.6) 0.63	1004 (5.5) 0.66	1004 (5.6) 0.66	2	1	δ(=C–H)
1027 (28.9) 0.22	1011 (29.8) 0.13	1012 (29.10) 0.13	17	16	ν(O_9_–CH_3_)
1042 (3.5) 0.74	1023 (6.2) 0.70	1023 (6.3) 0.71	19	19	ν(O_8_–CH_3_)
1092 (4.8) 0.17	1087 (5.4) 0.21	1088 (5.5) 0.21	6	6	ρ(=CH_2_)
1134 (16.2) 0.68	1130 (31.3) 0.44	1130 (29.3) 0.46	4	4	β(C–H) 8a
1139 (0.6) 0.75	1138 (1.2) 0.61	1138 (1.3) 0.64	1	1	ρ(CH_3_) a″
1139 (0.4) 0.75	1139 (1.2) 0.72	1139 (1.2) 0.73	1	1	ρ(CH_3_′) a″
1148 (41.14) 0.06	1141 (30.11) 0.05	1141 (31.14) 0.05	7	7	β(C–H)
1175 (1.6) 0.25	1174 (1.6) 0.17	1174 (1.7) 0.18	1	1	ρ(CH_3_′) a′
1180 (2.12) 0.40	1179 (2.7) 0.36	1179 (2.8) 0.37	1	1	ρ(CH_3_) a′
1203 (1.38) 0.39	1201 (3.22) 0.43	1201 (3.27) 0.43	2	2	ρ(–CH_2_)
1231 (91.1) 0.63	1218 (64.1) 0.41	1219 (65.2) 0.42	14	13	[ν(C_2_–O_12_)+ ν (C_1_–O_10_)]op
1255 (100.36) 0.06	1242 (100.50) 0.03	1242 (100.60) 0.03	13	13	[ν(C2–O12)+ ν(C1–O10)]ip7a
1269 (1.3) 0.57	1272 (1.3) 0.47	1272 (1.3) 0.47	−3	−3	β(C–H) 3
1287 (1.39) 0.26	1283 (1.34) 0.27	1283 (1.41) 0.27	4	4	δ(=C–H)
1296 (1.25) 0.39	1294 (0.8) 0.41	1295 (0.11) 0.41	2	2	ω(–CH_2_)
1340 (14.71) 0.09	1332 (10.52) 0.07	1333 (11.64) 0.07	8	7	ν(R) 14
1407 (12.5) 0.58	1404 (11.4) 0.20	1405 (11.4) 0.22	3	2	ν(R) 19b
1413 (3.20) 0.39	1408 (1.9) 0.44	1408 (1.12) 0.43	6	6	β_s_(=CH_2_)
1438 (9.8) 0.61	1435 (5.5) 0.75	1436 (5.6) 0.75	3	3	δ_s_(CH_3_′) a′
1439 (1.6) 0.75	1437 (5.4) 0.75	1437 (4.5) 0.75	2	2	β_s_(–CH_2_)
1447 (2.8) 0.49	1444 (1.7) 0.54	1444 (1.9) 0.54	3	3	δ_s_(CH_3_) a′
1454 (2.20) 0.75	1447 (3.10) 0.75	1447 (2.13) 0.75	7	6	δ_as_(CH_3_) a″
1454 (6.17) 0.75	1447 (4.9) 0.75	1448 (4.11) 0.75	8	7	δ_as_(CH_3_′) a″
1467 (20.7) 0.44	1459 (22.2) 0.23	1459 (22.2) 0.21	8	7	δ_as_(CH_3_) a′
1467 (15.10) 0.66	1460 (6.6) 0.75	1460 (6.8) 0.75	8	7	δ_as_(CH_3_′) a′
1508 (82.10) 0.28	1499 (66.7) 0.17	1500 (67.8) 0.18	8	8	ν(R) 19a
1582 (15.28) 0.75	1577 (12.27) 0.74	1578 (12.33) 0.74	5	5	ν(R) 8a
1600 (7.92) 0.62	1595 (4.68) 0.66	1595 (4.84) 0.66	5	5	ν(R) 8b
1653 (7.90) 0.13	1645 (7.64) 0.11	1646 (7.78) 0.12	7	7	ν(C=C)
2867 (35.60) 0.05	2883 (24.22) 0.04	2882 (25.29) 0.03	−15	−14	ν_s_(CH_3_) a′
2870 (13.100) 0.02	2885 (11.25) 0.05	2884 (11.51) 0.03	−14	−14	ν_s_(CH_3_) a′
2878 (11.83) 0.03	2885 (8.100) 0.01	2885 (9.100) 0.02	−7	−6	ν_s_(–CH_2_)
2911 (5.38) 0.73	2921 (6.32) 0.71	2920 (6.38) 0.71	−10	−10	ν_as_(–CH_2_)
2921 (15.35) 0.75	2943 (12.19) 0.75	2942 (12.23) 0.75	−21	−20	ν_as_(CH_3_) a″
2924 (16.24) 0.75	2945 (12.17) 0.75	2944 (12.20) 0.75	−20	−19	ν_as_(CH_3_) a″
2983 (6.26) 0.21	2980 (7.9) 0.32	2980 (7.11) 0.31	4	3	ν_s_(=CH_2_)
2991 (2.63) 0.20	2989 (2.47) 0.20	2989 (2.56) 0.20	3	2	ν(=C–H)
2993 (8.57) 0.48	3002 (6.24) 0.53	3001 (6.30) 0.53	−9	−8	ν_as_(CH_3_) a′
2994 (11.68) 0.41	3003 (7.26) 0.50	3002 (7.33) 0.50	−9	−8	ν_as_(CH_3_) a′
3026 (5.38) 0.36	3029 (4.28) 0.35	3029 (4.34) 0.35	−3	−3	ν(C–H) 7b
3056 (2.13) 0.20	3057 (1.17) 0.23	3057 (2.20) 0.23	−1	−1	ν(C–H) 20b
3063 (4.58) 0.21	3064 (6.20) 0.67	3064 (6.25) 0.67	−1	−1	ν(C–H) 2
3066 (7.42) 0.59	3068 (2.38) 0.22	3067 (2.47) 0.22	−2	−2	ν_as_(=CH_2_)

# Notations are similar to the Table S3.

**Table 7:** HOMO-LUMO energies and related quantities for ME.

Conformers	Energy in Unit (eV)			Electronegativity (χ)	Chemical Hardness (η)
	HOMO	LUMO	ΔE		
I	−5.6844	−0.3170	5.3674	3.0007	2.6837
II	−5.6447	−0.3474	5.2973	2.9961	2.6486
III	−5.6776	−0.3132	5.3644	2.9954	2.6822

**Table 8:** UV-vis absorption bands and the corresponding transitions for ME.

Absorption Bands		Excitation Energies (eV)	Oscillator Strength (f)	* Contributions
λ_exp_ (nm)	λ_cal_ (nm)			
265	247.7	5.0047	0.0723	H – 1 → L + 2 (3.4%) H – 1 → L + 4 (3.9%) H – 1 → L + 7 (2.57%) H − 1 → L + 10 (2.87) H → L (2.9%) H → L + 1 (21.98%) H →L + 2 (10.25%) H → L + 3 (45.41%) H → L + 4 (2.64%) H → L + 5 (4.14%)
219 *	223.4	5.5503	0.0902	H − 1 → L + 3 (3.50%) H → L (54.89%) H → L + 1 (5.63%) H → L + 2 (3.99%) H → L + (14.50%) H → L + 6 (7.42%) H → L + 8 (7.78%) H → L + 10 (2.29%)
219 *	220.7	5.6169	0.1474	H − 1 → L + 1 (1.82%) H − 1 → L + 3 (2.85%) H → L (7.84%) H → L + 2 (15.89%) H → L + 3 (6.77%) H → L + 4 (4.97%) H → L + 5 (1.30%) H → L + 7 (6.04%) H → L + 8 (2.60%) H → L + 9 (2.43%) H → L + 10 (47.49%)

* H stands for the HOMO, L stand for LUMO.

## Data Availability

The data supporting reported results can be found from one of the authors (R.K.Y.).

## Supplementary Materials

The following supporting information can be downloaded at: https://www.mdpi.com/article/10.3390/molecules28145409/s1, Figure S1: The optimised structures of all the higher energy conformers (C-IV to C-XXI). Figure S2. Total energy vs. dihedral angle curves for the tops (a) –^20^CH_3_, (b) –^24^CH_3_ and (c) C=C axes. Figure S3. Observed (a) IR and (b) Raman spectra of ME. Figure S4. Computed (a) IR and (b) Raman spectra of the 3 lower energy conformers of ME. Table S1. Distribution of the all possible conformers of ME according to the functional group orientations. Table S2. Geometrical parameters of ME for the 3 lower energy conformers. Table S3. Computed frequencies and related quantities for the 3 lower energy conformers of ME.
