# Supplementary material for: Experimental IR, Raman, and UV-Vis Spectra DFT Structural and Conformational Studies: Bioactivity and Solvent Effect on Molecular Properties of Methyl-Eugenol
Source: Molecules. 2023 Jul 14;28(14):5409. doi: 10.3390/molecules28145409 (PMC10386374; doi:10.3390/molecules28145409)
Supplement: Supplementary file 1 [file molecules-28-05409-s001.zip › molecules-2429768-supplementary.pdf]

Supplementary Materials

1. SM Figures.

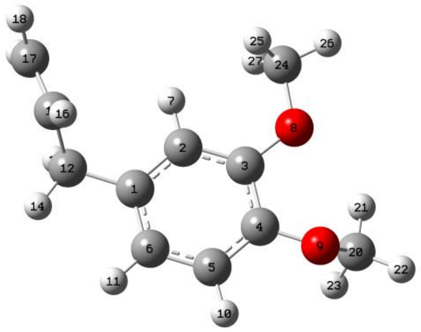

C-IV

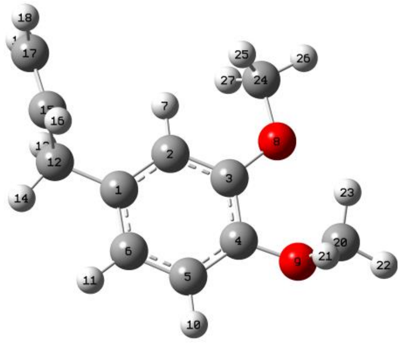

C-V

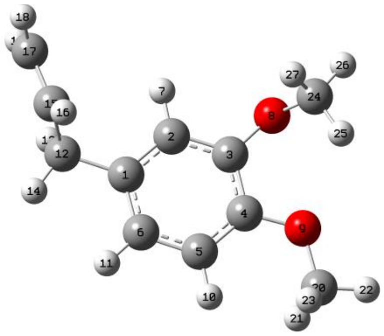

C-VI

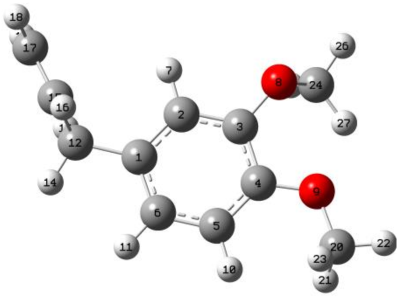

C-VII

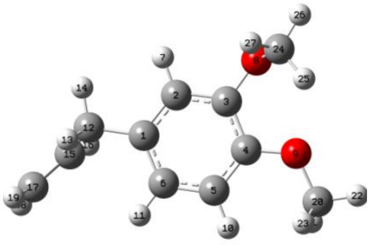

C-VIII

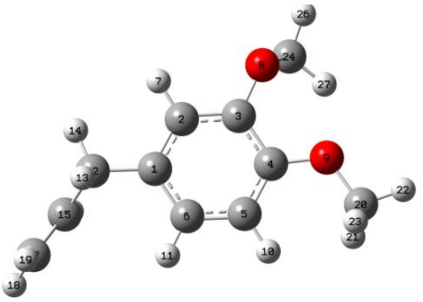

C-IX

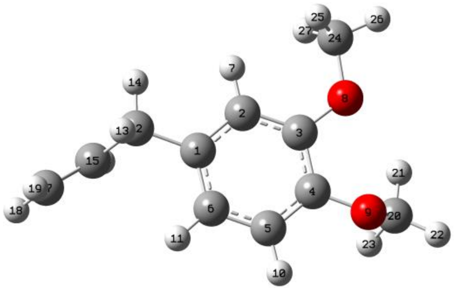

C-X

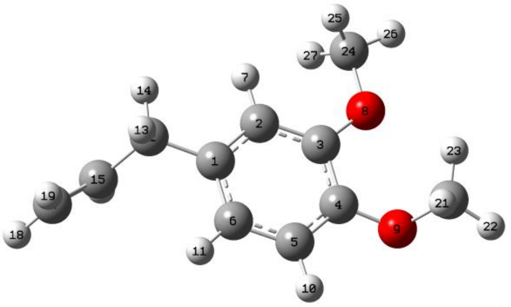

C-XI

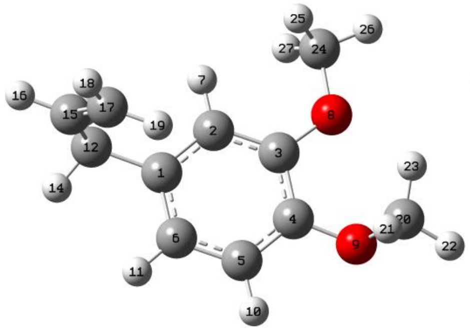

C-XII

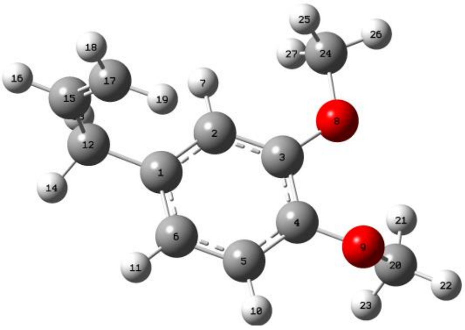

C-XIII

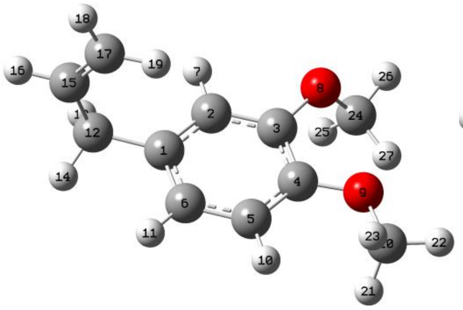

C-XIV

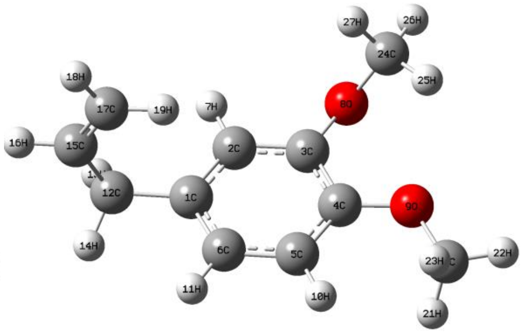

C-XV

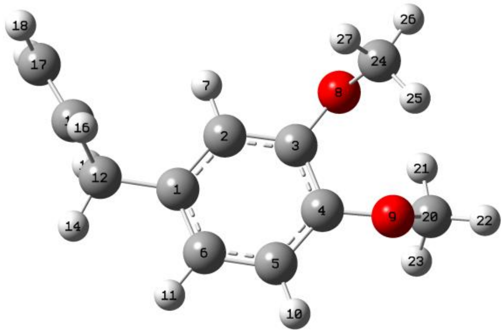

C-XVI

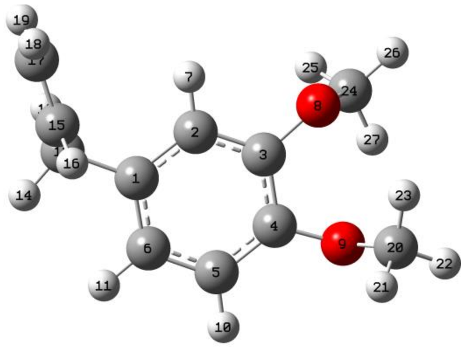

C-XVII

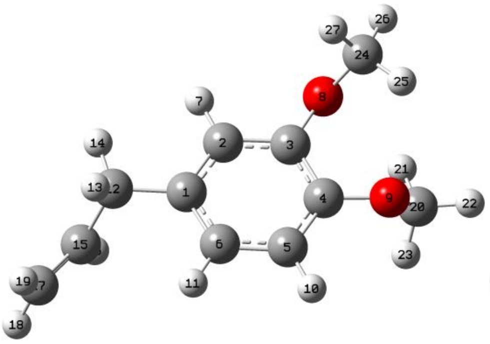

C-XVIII

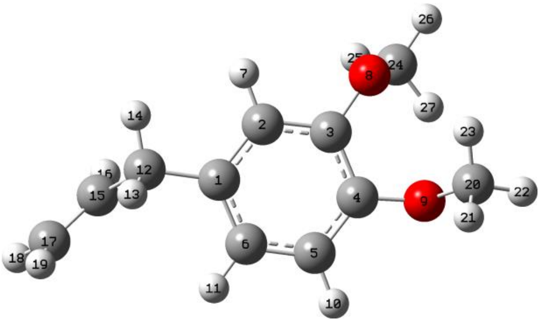

C-XIX

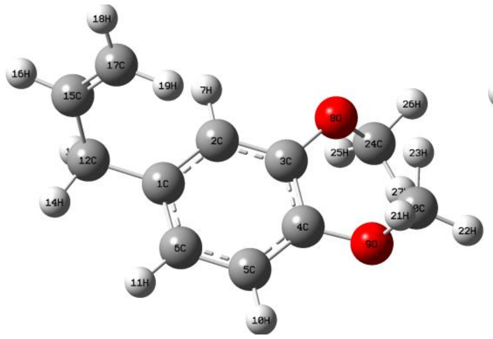

C-XX

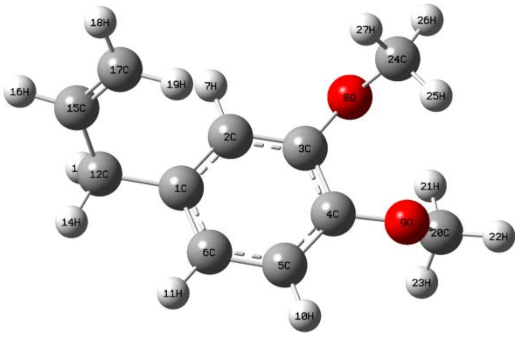

C-XXI

Figure S1: The optimised structures of all the higher energy conformers (C-IV to C-XXI).

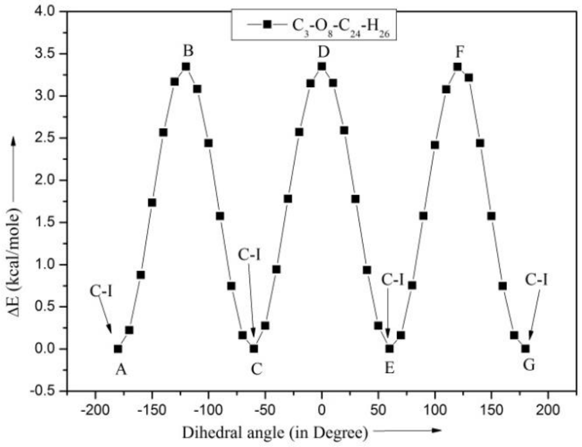

(a)

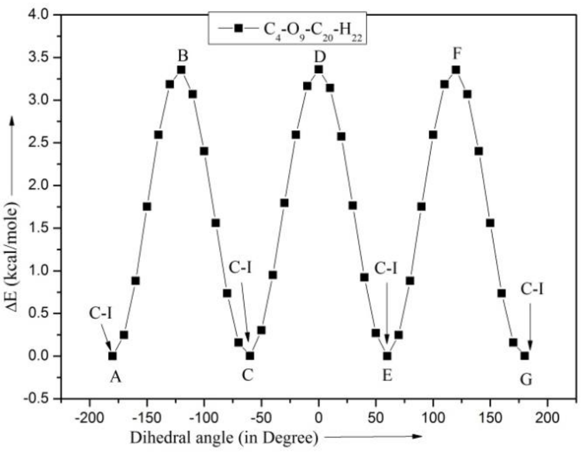

(b)

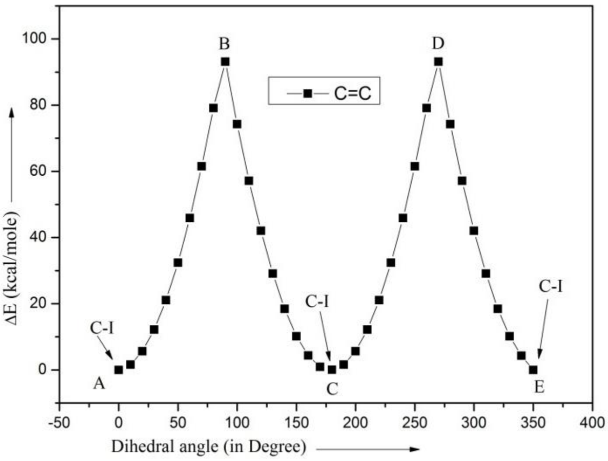

(c)

Figure S2. Total energy *vs* dihedral angle curves for the tops (a) -$^{20}CH_3$, (b) -$^{24}CH_3$ and (c) C=C axes.

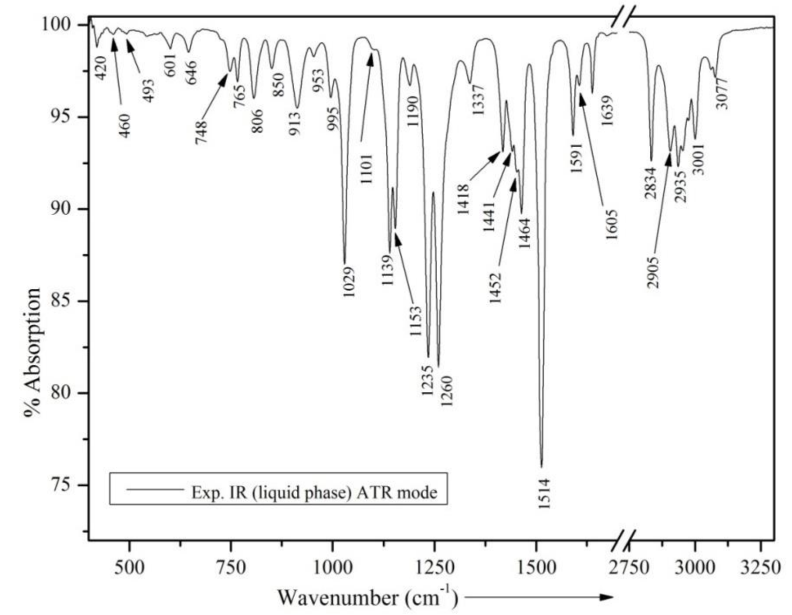

(a)

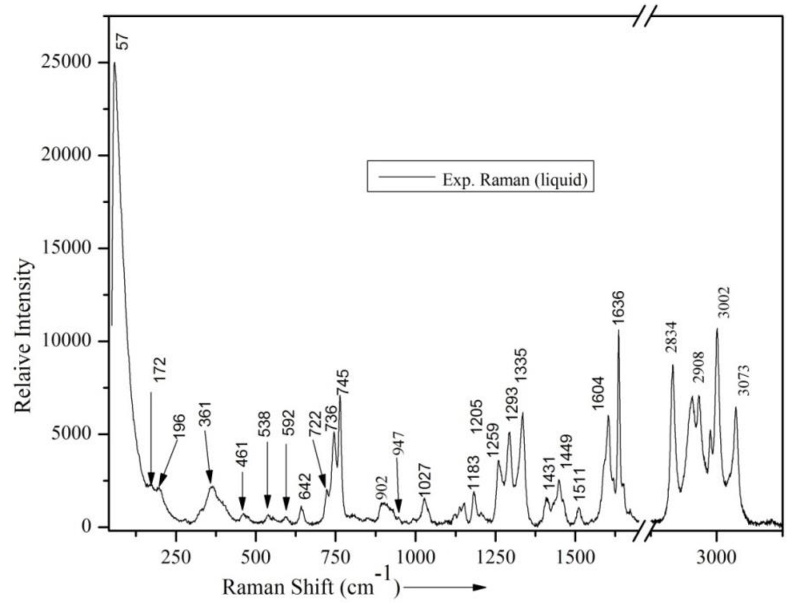

(b)

Figure S3. Observed (a) IR and (b) Raman spectra of ME.

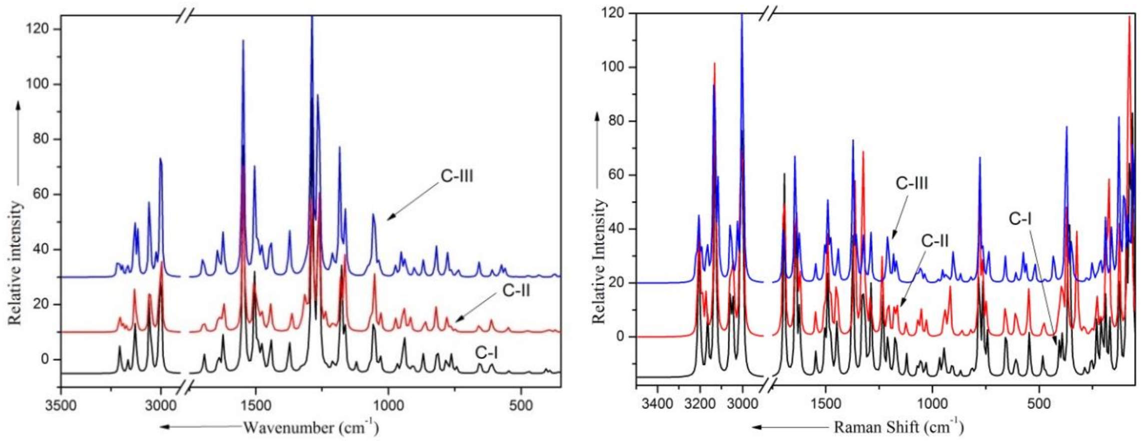

(a) (b)

Figure S4. Computed (a) IR and (b) Raman spectra of the 3 lower energy conformers of ME.

2. SM Tables

Table S1. Distribution of the all possible conformers of ME according to the functional group orientations.

Orientations of OCH_3 groups	Orientation of Allyl group		
	A_1	A_2	A_3
$M_{1U}\times M_{2D}$	C-I	C-II	C-III
$M_{1U}\times M_{2-}$	C-IV	C- X	C-XIII
$M_{1U}\times M_{2+}$	C-V	C-XI	C-XII
$M_{1+}\times M_{2D}$	C-VI	C-VIII	C-XV
$M_{1-}\times M_{2D}$	C-VII	C-IX	C-XIV
$M_{1+}\times M_{2-}$	C-XVI	C-XVIII	C-XX
$M_{1-}\times M_{2+}$	C-XVII	C-XIX	C-XXI

M_1 and M_2 are the methoxy groups at the meta- and para-positions relative to the allyl group, The symbols U and D indicate that the CH_3 group is up and down in the ring plane itself and signs + and – indicate that the CH3 group is above and below the ring plane.

Table S2. Geometrical parameters of ME for the 3 lower energy conformers.

Parameters[#]	C-I	C-II	C-III	C-I	
				Water	Ethenol
$r(C_1\text{-}C_2)$	1.406	1.405	1.404	1.406	1.406
$r(C_1\text{-}C_6)$	1.387	1.388	1.390	1.389	1.389
$r(C_1\text{-}C_{12})$	1.520	1.512	1.521	1.520	1.520
$r(C_2\text{-}C_3)$	1.389	1.390	1.392	1.390	1.390
$r(C_2\text{-}H_7)$	1.082	1.083	1.083	1.082	1.082
$r(C_3\text{-}C_4)$	1.418	1.417	1.415	1.418	1.418
$r(C_3\text{-}O_8)$	1.361	1.362	1.362	1.364	1.364
$r(C_4\text{-}C_5)$	1.389	1.390	1.391	1.390	1.390
$r(C_4\text{-}O_9)$	1.362	1.362	1.362	1.365	1.365
$r(C_5\text{-}C_6)$	1.402	1.400	1.399	1.402	1.402
$r(C_5\text{-}H_{10})$	1.082	1.082	1.082	1.082	1.082
$r(C_6\text{-}H_{11})$	1.085	1.085	1.084	1.085	1.085
$r(O_8\text{-}C_{24})$	1.420	1.419	1.419	1.428	1.428
$r(O_9\text{-}C_{20})$	1.419	1.419	1.419	1.428	1.428
$r(C_{12}\text{-}H_{13})$	1.096	1.098	1.096	1.095	1.095
$r(C_{12}\text{-}H_{14})$	1.096	1.097	1.097	1.096	1.096
$r(C_{12}\text{-}C_{15})$	1.507	1.513	1.506	1.508	1.508
$r(C_{15}\text{-}H_{16})$	1.089	1.089	1.089	1.089	1.089
$r(C_{15}\text{-}C_{17})$	1.332	1.331	1.331	1.333	1.333
$r(C_{17}\text{-}H_{18})$	1.084	1.084	1.084	1.085	1.085
$r(C_{17}\text{-}H_{19})$	1.086	1.085	1.086	1.086	1.086
$r(C_{20}\text{-}H_{21})$	1.096	1.096	1.096	1.094	1.094
$r(C_{20}\text{-}H_{22})$	1.089	1.089	1.089	1.088	1.088
$r(C_{20}\text{-}H_{23})$	1.096	1.096	1.096	1.094	1.094
$r(C_{24}\text{-}H_{25})$	1.096	1.096	1.096	1.094	1.094
$r(C_{24}\text{-}H_{26})$	1.089	1.089	1.089	1.088	1.088
$r(C_{24}\text{-}H_{27})$	1.096	1.096	1.096	1.094	1.094
$\alpha(C_2\text{-}C_1\text{-}C_6)$	118.5	118.4	118.4	118.5	118.5
$\alpha(C_2\text{-}C_1\text{-}C_{12})$	120.0	120.3	120.4	120.0	120.0
$\alpha(C_6\text{-}C_1\text{-}C_{12})$	121.5	121.4	121.2	121.5	121.5
$\alpha(C_1\text{-}C_2\text{-}C_3)$	121.4	121.5	121.6	121.3	121.4
$\alpha(C_1\text{-}C_2\text{-}H_7)$	118.7	118.8	118.8	118.9	118.9
$\alpha(C_3\text{-}C_2\text{-}H_7)$	119.9	119.7	119.6	119.8	119.8
$\alpha(C_2\text{-}C_3\text{-}C_4)$	119.6	119.5	119.5	119.6	119.6
$\alpha(C_2\text{-}C_3\text{-}O_8)$	124.8	124.8	124.7	124.7	124.7
$\alpha(C_4\text{-}C_3\text{-}O_8)$	115.6	115.7	115.8	115.7	115.7
$\alpha(C_3\text{-}C_4\text{-}C_5)$	119.0	119.0	119.0	119.1	119.1
$\alpha(C_3\text{-}C_4\text{-}O_9)$	115.9	115.9	116.0	116.0	116.0
$\alpha(C_5\text{-}C_4\text{-}O_9)$	125.1	125.1	125.1	124.9	125.0
$\alpha(C_4\text{-}C_5\text{-}C_6)$	120.7	120.7	120.8	120.6	120.6

α(C_4-C_5-H_{10})	120.2	120.1	120.1	120.2	120.2
α(C_6-C_5-H_{10})	119.2	119.2	119.1	119.2	119.2
α(C_1-C_6-C_5)	120.9	120.9	120.8	120.9	120.9
α(C_1-C_6-H_{11})	120.1	120.0	120.0	120.1	120.1
α(C_5-C_6-H_{11})	119.1	119.1	119.2	119.0	119.0
α(C_3-O_8-C_{24})	118.3	118.4	118.4	118.3	118.3
α(C_4-O_9-C_{20})	118.2	118.2	118.2	118.1	118.1
α(C_1-C_{12}-H_{13})	110.1	109.7	110.0	110.1	110.1
α(C_1-C_{12}-H_{14})	108.3	109.3	108.5	108.3	108.3
α(C_1-C_{12}-C_{15})	113.6	116.2	113.6	113.2	113.2
α(H_{13}-C_{12}-H_{14})	106.5	105.0	106.4	106.9	106.9
α(H_{13}-C_{12}-C_{15})	109.1	108.0	109.2	109.2	109.2
α(H_{14}-C_{12}-C_{15})	109.0	108.0	108.9	109.0	109.0
α(C_{12}-C_{15}-H_{16})	115.6	114.4	115.6	115.6	115.6
α(C_{12}-C_{15}-C_{17})	125.1	126.8	125.1	125.1	125.1
α(H_{16}-C_{15}-C_{17})	119.3	118.8	119.3	119.3	119.3
α(C_{15}-C_{17}-H_{18})	121.6	121.0	121.6	121.6	121.6
α(C_{15}-C_{17}-H_{19})	121.6	121.9	121.6	121.5	121.5
α(H_{18}-C_{17}-H_{19})	116.8	117.1	116.8	116.9	116.9
α(O_9-C_{20}-H_{21})	111.5	111.5	111.5	111.3	111.3
α(O_9-C_{20}-H_{22})	105.8	105.8	105.8	105.8	105.8
α(O_9-C_{20}-H_{23})	111.5	111.5	111.5	111.2	111.2
α(H_{21}-C_{20}-H_{22})	109.3	109.3	109.3	109.5	109.5
α(H_{21}-C_{20}-H_{23})	109.3	109.3	109.3	109.6	109.6
α(H_{22}-C_{20}-H_{23})	109.3	109.3	109.3	109.5	109.5
α(O_8-C_{24}-H_{25})	111.5	111.5	111.5	111.2	111.2
α(O_8-C_{24}-H_{26})	105.7	105.7	105.7	105.7	105.7
α(O_8-C_{24}-H_{27})	111.5	111.5	111.5	111.2	111.3
α(H_{25}-C_{24}-H_{26})	109.4	109.3	109.3	109.5	109.5
α(H_{25}-C_{24}-H_{27})	109.3	109.4	109.4	109.6	109.6
α(H_{26}-C_{24}-H_{27})	109.3	109.3	109.3	109.5	109.5

[#] Geometrical parameter bond length (r) in (Å), bond angles (º).

Table S3. Computed frequencies and related quantities for the 3 lower energy conformers of ME.

*C-I	C-II	C-III	†IR	†Raman	#PEDs	Assignment
28(0,29)0.74	20(0,54)0.74	28(0,332)0.75	-	-	$\tau(C_1-C_{12})(69)+\gamma(C_1-C_{12})(15)+\alpha(C_1-C_{12}-C_{15})(6)$	$\tau(C_1-C_{12})$
64(1,95)0.69	65(2,24)0.73	59(2,45)0.69	-	57 (vs)	$\tau(C_4-O_9)(24)+\tau(C_{12}-C_{15})(23)+\alpha(C_1-C_{12}-C_{15})(9)-\tau(O_9-C_{20})(9)-\tau(C_3-O_8)(6)+\varphi(R)(5)+\beta(C_1-C_{12})(4)+\delta 2(C_{15}-H_{16})(4)$	$\tau(C_4-OCH_3)$
76(2,48)0.71	75(2,55)0.74	81(2,14)0.72	-	-	$\tau(C_4-O_9)(29)-\tau(C_3-O_8)(25)-\tau(C_{12}-C_{15})(15)-\tau(O_9-C_{20})(9)+\tau(O_8-C_{24})(9)-\alpha(C_1-C_{12}-C_{15})(4)$	$\tau(C_{12}-C_{15})$
89(0,40)0.74	82(1,37)0.72	100(0,32)0.65	-	-	$\tau(C_3-O_8)(47)-\tau(O_8-C_{24})(18)+\tau(C_4-O_9)(11)+\varphi(R)(7)-\tau(O_9-C_{20})(5)-\tau(C_1-C_{12})(5)$	$\tau(C_3-OCH_3)$
121(0,36)0.75	121(0,23)0.75	125(0,42)0.75	-	-	$\tau(C_4-O_9)(31)-\varphi(R)(18)+\tau(C_{12}-C_{15})(13)-\gamma(C_1-C_{12})(11)-\tau(O_9-C_{20})(6)+\tau(C_3-O_8)(5)-\alpha(C_1-C_{12}-C_{15})(3)$	$\gamma(C_1-C_{12})$
165(0,17)0.73	170(0,36)0.75	157(0,14)0.52	-	- 170(m)	$\varphi(R)(26)+\varphi(R)(30)+\gamma(C_1-C_{12})(10)-\tau(C_3-O_8)(6)-\alpha(C_1-C_{12}-C_{15})(5)+\tau(C_{12}-C_{15})(5)-\tau(C_4-O)(4)$	$\gamma(C_3-O_8)$
186(2,23)0.50	181(2,29)0.45	185(2,16)0.49	-	196 (m)	$\beta(C_3-O_8)(35)-\beta(C_4-O_9)(24)+\alpha(C_3-O_8-C_{24})(10)-\alpha(R)(5)+\alpha(C_4-O_9-C_{20})(5)+\varphi(R)(3)$	$\beta(C_3-O_8)$
204(0,21)0.62	205(0,4)0.73	209(0,8)0.60	-	-	$\beta(C_1-C_{12})(23)+\beta(C_4-O_9)(20)-\alpha(C_4-O_9-C_{20})(12)-\tau(C_{12}-C_{15})(7)-\tau(O_8-C_{24})(6)+\varphi(R)(5)+\alpha(C_3-O_8-C_{24})(4)$	$\gamma(C_4-O_9)$
225(0,18)0.69	226(0,12)0.72	223(0,2)0.72	-	-	$\tau(O_8-C_{24})(32)-\tau(O_9-C_{20})(18)-\alpha(C_1-C_{12}-C_{15})(10)-\varphi(R)(11)-\gamma(C_1-C_{12})(5)$	$\tau(OCH_3)$
250(0,7)0.68	250(0,2)0.57	245(0,6)0.61	-	-	$\tau(O_9-C_{20})(62)+\tau(O_8-C_{24})(13)+\varphi(R)(9)-\alpha(C_1-C_{12}-C_{15})(4)$	$\tau(OCH_3)$
279(0,3)0.33	283(0,3)0.40	271(0,1)0.18	-	276 (w)	$\tau(O_8-C_{24})(35)+\varphi(R)(22)+\gamma(C_2-H_7)(9)+\alpha(C_1-C_{12}-C_{15})(9)$	$\alpha(C_1-C_{12}-C_{15})$
345(0,18)0.25	316(0,26)0.15	340(1,13)0.41	-	-	$\beta(C_1-C_{12})(25)-\alpha(C_3-O_8-C_{24})(20)-\alpha(C_{14}-C_{15}-C_{17})(12)+\alpha(C_4-O_9-C_{20})(7)-\alpha(R)(5)-\varphi(R)(4)-\gamma(C_1-C_{12})(3)-\beta(C_4-O_9)(3)$	$\beta(C_1-C_{12})$
354(0,74)0.11	363(1,32)0.13	364(1,36)0.11	-	361 (m)	$\alpha(C_4-O_9-C_{20})(13)-\alpha(R)(13)+\alpha(C_{14}-C_{15}-C_{17})(11)+\alpha(C_3-O_8-C_{24})(8)-\varphi(R)(5)+\beta(C_1-C_{12})(4)+\beta(C_4-O_9)(4)+\nu(C_1-C_{12})(4)-\gamma(C_4-O_9)(3)+\nu(C_3-C_4)(3)$	$\beta(C_1-O_{10})$
384(1,12)0.22	385(2,18)0.18	369(1,14)0.19	-	-	$\alpha(C_4-O_9-C_{20})(23)-\alpha(C_{14}-C_{15}-C_{17})(19)+\alpha(C_3-O_8-C_{24})(16)-\beta(C_3-O_8)(6)+\nu(C_3-C_4)(4)+\alpha(R)(4)-\nu(C_1-C_{12})(3)$	$\alpha(C-O-C)_{ipc}$
398(2,10)0.72	399(1,8)0.59	423(1,10)0.75	- 420(m) ##	403 (w) -	$\alpha(C_{14}-C_{15}-C_{17})(15)+\gamma(C_4-O_9)(15)+\varphi(R)(19)-\alpha(C_1-C_{12}-C_{15})(10)+\beta(C_1-H_{12})(6)+\gamma(C_5-H_{10})(5)-\tau(O_9-C_{20})(5)+\gamma(C_1-H_{12})(4)+\gamma(C_3-O_8)(3)-\gamma(C_6-H_{11})(3)$	$\alpha(C_{14}-C_{15}=C_{17})$
462(1,2)0.69	463(1,1)0.73	463(1,1)0.69	460(w)	461(w)	$\varphi(R)(45)-\gamma(C_3-O_8)(21)-\gamma(C_4-O_9)(20)$	$\Phi(R)$ 16b
474(0,6)0.61	470(1,4)0.75	- 510(0,6)0.55	483(w)	473(w)	$\alpha(R)(27)+\alpha(C_{14}-C_{15}-C_{17})(12)-\varphi(R)(8)+\alpha(C_4-O_9-C_{20})(4)+\alpha(C_1-C_{12}-C_{15})(4)-t(CH_2)(4)-\nu(C_4-O_9)(3)$	$\alpha(R)$ 6a
534(1,15)0.52	538(3,13)0.46	549(3,4)0.75	542(w)	538w 553(w) ##	$\alpha(R)(32)-\alpha(C_3-O_8-C_{24})(14)+\nu(C_3-O_8)(8)+\nu(C_1-C_{12})(6)+\nu(C_1-C_2)(6)-\alpha(R)(5)-\delta_2(C_{15}-H_{16})(5)+\tau(C_{15}=C_{17})(4)-\alpha(C_{14}-C_{15}-C_{17})(3)$	$\alpha(R)$ 6b
592(1,5)0.54	589(1,5)0.75	564(4,9)0.55	-	592(w)	$\beta(C_4-O_9)(18)+\alpha(C_4-O_9-C_{20})(15)+\beta(C_3-O_8)(14)-\alpha(C_3-O_8-C_{24})(9)+\gamma(C_1-C_{12})(6)-\nu(C_4-C_5)(5)-\beta(C_1-C_{12})(4)-\varphi(R)(8)$	$\alpha(C-O-C)_{opc}$
600(4,6)0.72	599(7,5)0.35	596(2,3)0.54	601(w)	596(w)	$\gamma(C_1-C_{12})(21)-\varphi(R)(34)-\delta_2(C_{15}-H_{16})(10)+\tau(C_{15}=C_{17})(8)+\gamma(C_3-O_8)(6)$	$\delta(=C-H)$
642(6,22)0.49	644(5,11)0.47	645(5,7)0.52	646(w)	647(w)	$\alpha(R)(23)+\gamma(C_1-C_{12})(10)-\varphi(R)(8)+\delta_2(C_{15}-H_{16})(8)-\tau(C_{15}=C_{17})(7)-\nu(C_1-C_{12})(5)+\gamma(C_3-O_8)(5)-\nu(C_{12}-C_{15})(4)+\nu(C_4-O_9)(3)$	$\alpha(R)$ 12
728(2,15)0.17	732(1,7)0.17	723(3,11)0.08	724(w)	722m	$\varphi(R)(38)+\gamma(C_3-O_8)(19)-\gamma(C_4-O_9)(15)-\gamma(C_6-H_{11})(7)$	$\Phi(R)$ 4
748(7,30)0.13	751(3,21)0.17	748(2,8)0.09	748(m)	745ms	$\varphi(R)(29)-\gamma(C_4-O_9)(10)+\alpha(R)(9)+\gamma(C_3-O_8)(7)-\nu(C_3-O_8)(5)+\alpha(C_1-C_{12}-C_{15})(5)-\gamma(C_1-H_{12})(5)-\nu(C_3-C_4)(4)-\delta_2(C_{15}-H_{16})(3)+\tau(C_{15}=C_{17})(3)$	$\Phi(R)$ 16a
766(6,47)0.07	764(8,37)0.08	762(8,37)0.09	765(m)	766s	$\nu(C_4-O_9)(19)+\alpha(R)(23)+\nu(C_3-C_4)(8)+\nu(C_4-C_5)(7)+\nu(O_9-C_{20})(5)-\alpha(C_4-O_9-C_{20})(5)-\alpha(C_3-O_8-C_{24})(3)$	$\nu(R)$ 1
799(12,3)0.24	804(13,1)0.57	804(11,1)0.46	806(m)	805m	$\gamma(C_5-H_{10})(39)+\gamma(C_6-H_{11})(24)-\gamma(C_4-O_9)(12)-\varphi(R)(13)+\gamma(C_3-O_8)(3)$	$\gamma(C-H)$
851(7,2)0.13	846(8,2)0.38	850(5,2)0.19	850(m)	853w	$\gamma(C_2-H_7)(61)-\varphi(R)(17)-\gamma(C_3-O_8)(6)$	$\gamma(C_2-H_7)$
		883(4,11)0.04	-		$t(CH_2)(27)-\varrho(=CH_2)(15)-\gamma(C_5-H_{10})(12)-\gamma(C_2-$	$t(C_{12}-H)$

888(4,6)0.14	899(6,7)0.03			902(m)	H$_7$)(11)+γ(C$_6$-H$_{11}$)(6)-δ$_1$(C$_{15}$-H$_{16}$)(5)	
903(1,1)0.44	901(2,10)0.06	909(0,1)0.11	-	-	γ(C$_6$-H$_{11}$)(43)-γ(C$_5$-H$_{10}$)(27)-φ(R)(13)-t(CH$_2$)(7)	γ(C-H)
920(12,3)0.47	913(1,1)0.37	917(6,2)0.63	913(m)	923(m)	ω(=CH$_2$)(55)+ν(C$_{12}$-C$_{15}$)(21)+ ϱ(=CH$_2$)(9)+t(CH$_2$)(3)	ω(=CH$_2$)
928(6,11)0.48	924(19,10)0.43	932(8,4)0.74	-	-	ω(=CH$_2$)(44)-ν(C$_{12}$-C$_{15}$)(24)-ϱ(=CH$_2$)(9)-t(CH$_2$)(5)+α(C$_1$-C$_{12}$-C$_{15}$)(3)+τ(C$_{15}$=C$_{17}$) (3)	ν(C$_{12}$-C$_{15}$)
948(3,7)0.05	953(7,0)0.75	954(3,1)0.66	953(m)	947(w) 965(w)	ν(C$_1$-C$_{12}$)(16)-ν(C$_3$-O$_8$)(11)-ν(O$_8$-C$_{24}$) (11)+ν(C$_1$-C$_2$)(10)+t(CH$_2$)(7) +α(R)(7)-ϱ(=CH$_2$)(5)-β(C$_3$-O$_8$)(4)+ ν(C$_1$-C$_6$)(4)	ν(C$_1$-C$_{12}$) 20a
1005(6,6)0.63	1003(9,4)0.56	1008(5,3)0.75	995(m)	994(m)	τ(C$_{15}$=C$_{17}$)(55)+δ$_2$(C$_{15}$-H$_{16}$)(39)	τ(C=C)
1027(28,9)0.22	1027(35,8)0.20	1027(32,6)0.18	1029(ms)	1027(m)	ν(O$_9$-C$_{20}$)(43)-α(R)(27)-ν(O$_8$-C$_{24}$)(16)	ν(O$_9$-CH$_3$)
1042(3,5)0.74	1042(5,4)0.72	1041(3,4)0.65	-	1039(m)sh	ν(O$_8$-C$_{24}$)(47)+ν(O$_9$-C$_{20}$)(28)-ν(C$_3$-C$_4$)(6)-ν(C$_4$-C$_5$)(3)	ν(O$_8$-CH$_3$)
1092(4,8)0.17	1094(0,4)0.40	1053(3,3)0.17	- 1101(ms)	-	ϱ(=CH$_2$)(22)+ϱ(CH$_2$)(20)-ν(C$_{12}$-C$_{15}$)(17) +δ$_1$(C$_{15}$-H$_{16}$)(10)+α(C$_{14}$-C$_{15}$-C$_{17}$)(7) +t(CH$_2$)(7)+α(C$_1$-C$_{12}$-C$_{15}$)(4)	ϱ(=CH$_2$)
1134(16,2)0.68	1135(38,4)0.20	1134(22,1)0.49	-	1122(m)	β(C$_6$-H$_{11}$)(29)+ν(C$_5$-C$_6$)(24)-β(C$_5$-H$_{10}$) (20)+β(C$_4$-O$_9$)(3)+ϱ1(CH$_3$)(3)-α(R)(3)	β(C-H)
1139(0,6)0.75	1138(2,5)0.72	1138(0,5)0.75	1139(m)	1138(m)	ϱ(CH$_3$)′(67)+ϱ2(CH$_3$)′(25)+δ$_{as}$1(CH$_3$)′(4)	ϱ(CH$_3$)a″
1139(0,4)0.75	1139(0,2)0.75	1138(0,2)0.75	1139(m)	1138(m)	ϱ(CH$_3$)(65)+ϱ2(CH$_3$)(26)+δ$_{as}$1(CH$_3$)(4)	ϱ(CH$_3$′)a″
1148(41,14)0.06	1149(30,8)0.12	1153(41,8)0.11	1153(ms)	1152(m)	ν(C$_1$-C$_{12}$)(15)-β(C$_2$-H$_7$)(14)-ν(C$_3$-O$_8$)(11)+ν(O$_8$-C$_{24}$)(9)-ν(R)(14)+ν(C$_4$-O$_9$)(5)-ν(O$_9$-C$_{20}$)(4)+β(C$_6$-H$_{11}$)(4)+ϱ$_2$(CH$_3$)(4)+α(R)(3)	β(C-H)
1175(1,6)0.25	1175(1,6)0.25	1174(2,6)0.25	-	-	ϱ$_2$(CH$_3$)(47)-ϱ$_1$(CH$_3$)(16)+ϱ$_2$(CH$_3$)′(8)-β(C$_5$-H$_{10}$)(4)-ν(C$_4$-C$_5$)(4)-δ$_{as}$2(CH$_3$)(4)	ϱ(CH$_3$′)a′
1180(2,12)0.40	1179(4,7)0.45	1180(0,3)0.75	1190(m)	1183(m)	ϱ$_2$(CH$_3$)′(45)-ϱ$_1$(CH$_3$)′(15)+ν(C$_1$-C$_{12}$)(9)+α(R)(5)-δ$_{as}$(CH$_3$)′(4)+α(C$_3$-O$_8$-C$_{24}$)(3)-ϱ$_2$(CH$_3$)(3)	ϱ(CH$_3$)a′
1203(1, 38)0.39	1205(8,21)0.59	1182(6,11)0.37	-	- 1206(m)	ϱ(CH$_2$)(52)-ϱ(=CH$_2$)(9)+ν(C$_{12}$-C$_{15}$)(8)-ν(C$_1$-C$_6$)(6)+ν(C$_1$-C$_2$)(4)+β(C$_6$-H$_{11}$)(4)-α(C$_{14}$-C$_{15}$-C$_{17}$)(3)-t(CH$_2$)(3)	ϱ(-CH$_2$)
1231(91,1)0.63	1231(99,0)0.45	1232(89,0)0.28	1235(s)	-	ν(C$_4$-O$_9$)(24)-α(R)(16)-ν(C$_3$-O$_8$)(9)-ν(O$_9$-C$_{20}$)(7)+β(C$_2$-H$_7$)(6)-β(C$_6$-H$_{11}$)(6)+ν(O$_8$-C$_{24}$)(3)	[ν(C$_2$-O$_{12}$)+ν(C$_1$-O$_{10}$)]$_{op}$
1255(100,36)0.06	1257(100,14)0.05	1257(100,18)0.03	1260(s)	1259(m)	ν(C$_3$-O$_8$)(16)-ν(C$_5$-C$_6$)(14)-ν(C$_3$-C$_4$)(13)+ν(C$_4$-O$_9$)(11)-ν(C$_1$-C$_2$)(10)-β(C$_5$-H$_{10}$)(9)-ν(O$_8$-C$_{24}$)(4)	[ν(C$_2$-O$_{12}$)+ν(C$_1$-O$_{10}$)]$_{ip}$7a
1269(1,3)0.57	1277(6,2)0.18	1279(3,1)0.74	-	-	ω(CH$_2$)(36)+β(C$_2$-H$_7$)(21)+β(C$_5$-H$_{10}$)(9)-δ$_1$(C$_{15}$-H$_{16}$)(6)+β(C$_6$-H$_{11}$)(5)	β(C-H) 3
1287(1,39)0.26	1282(16,4)0.75	1292(1,17)0.13	1285(m)	-	δ$_1$(C$_{15}$-H$_{16}$)(39)+β(C$_5$-H$_{10}$)(8)+ν(C$_{15}$=C$_{17}$) (7)+β(C$_2$-H$_7$)(7)-ϱ(=CH$_2$)(6)-ω(CH$_2$)(6)+ β(C$_6$-H$_{11}$)(5)	δ(=C-H)
1296(1,25)0.39	1294(6,51)0.21	1322(0,16)0.46	-	1293(m)	ω(CH$_2$)(35)+δ$_1$(C$_{15}$-H$_{16}$)(13)-ν(C$_1$-C$_{12}$)(6)+ν(C$_{15}$=C$_{17}$)(6)-β(C$_5$-H$_{10}$)(6)-βs(=CH$_2$)(4)-ν(C$_4$-C$_5$)(4)-β(C$_6$-H$_{11}$)(3)	ω(-CH$_2$)
340(14,71)0.09	1331(10,33)0.09	1339(16,50)0.19	1337(m)	1335(ms)	ν(R)(66)+ω(CH$_2$)(5)+ϱ(CH$_2$)(3)-ν(C$_4$-O$_9$)(3)-ν(C$_1$-C$_{12}$) (3)	ν(R)14
1407(12,5)0.58	1407(14,4)0.35	1405(6,9)0.73	1418(m)	1412(w)	β(C$_6$-H$_{11}$)(17)+ ν(C$_2$-C$_3$)(16)+δ$_s$(CH$_3$)′ (15)-ν(C$_5$-C$_6$)(10)+ν(C$_1$-C$_2$)(6)-ν(C$_3$-O$_8$)(4)-β(C$_1$-H$_{12}$)(4)-βs(-CH$_2$)(3)-β(C$_3$-O$_8$)(3)+β$_s$(=CH$_2$) (3)-ϱ(CH$_2$)(3)	ν(R)19b
1413(3,20)0.39	1414(3,11)0.47	1410(11,5)0.25	1418(m)	1412(m)	β$_s$(=CH$_2$) (65)+δ1(C$_{15}$-H$_{16}$)(12)+ν(C$_{12}$-C$_{15}$)(4)-β$_s$(-CH$_2$)(4)+ω(CH$_2$)(3)	β$_s$(=CH$_2$)
1438(9,8)0.61	1438(8,4)0.62	1437(5,4)0.61	1441(m)	1449(m)	δ$_s$(CH$_3$)(44)+β$_s$(-CH$_2$)(30)+δ$_s$(CH$_3$)′(13)	δ$_s$(CH$_3$′) a′
1439(1,6)0.75	1441(2,4)0.75	1439(5,9)0.62	1441(m)	1449(m)	β$_s$(-CH$_2$)(50)-δ$_s$(CH$_3$)(38)	β$_s$(-CH$_2$)
1447(2,8)0.49	1447(3,2)0.38	1447(2,3)0.27	1441(m)	1449(m)	δ$_s$(CH$_3$)′(61)-β$_s$(-CH$_2$)(10)- ν(C$_1$-C$_2$)(5)+ ν(C$_3$-O$_8$)(5)-β(C$_6$-H$_{11}$)(4)-δ$_s$(CH$_3$)(4)	δ$_s$(CH$_3$) a′
1454(2,20)0.75	1454(3,11)0.75	1454(3,14)0.75	1452(m)	1452sh	δ$_{as}$(CH$_3$)′(58)+δ$_{as}$(CH$_3$)′(26)+δ$_{as}$(CH$_3$)(6)-ϱ1(CH$_3$)′(5)	δ$_{as}$(CH$_3$) a″
1454(6,17)0.75	1455(5,13)0.75	1454(5,11)0.75	1452(ms)	1452(m)sh	δ$_{as}$(CH$_3$)(58)+δ$_{as}$2(CH$_3$)(25)-δ$_{as}$(CH$_3$)′(6)-ϱ1(CH$_3$)(5)	δ$_{as}$(CH$_3$′) a″
1467(20,7)0.44	1467(20,6)0.58	1466(20,5)0.50	1464(ms)	1461(m)	δ$_{as}$(CH$_3$)′(58)-δ$_{as}$(CH$_3$)′(26)+ϱ2(CH$_3$)′(9)	δ$_{as}$(CH$_3$) a′
1467(15,10)0.66	1467(21,6)0.54	1467(16,6)0.64	1464(ms)	1461(m)	δ$_{as}$(CH$_3$)(59)-δas(CH$_3$)(26)+ϱ2(CH$_3$)(9)	δ$_{as}$(CH$_3$′) a′
1508(82,10)0.28	1508(96,8)0.30	1509(80,6)0.28	1514(s)	1511(m)m	β(C$_5$-H$_{10}$)(17)-β(C$_2$-H$_7$)(14)-ν(C$_3$-C$_4$)(20)+ν(C$_4$-O$_9$)(10)-ν(C$_4$-C$_5$)(9)+ ν(C$_3$-O$_8$)(5)+δ$_s$(CH$_3$)(4)+β(C$_6$-H$_{11}$)(4)-β(C$_3$-O$_8$)(3)+ν(C$_5$-C$_6$)(3)	ν(R)19a
1582(15,28)0.75	1581(18,21)0.65	1582(16,16)0.71	1591(ms)	1591(m)sh	ν(C$_4$-C$_5$)(23)-ν(C$_1$-C$_6$)(16)+ν(C$_1$-C$_2$)(14)-ν(C$_3$-C$_4$)(12)-α(R)(9)+β(C$_4$-O$_9$)(4)-β(C$_5$-H$_{10}$)(4)	ν(R)8a
1600(7,92)0.62	1599(12,67)0.62	1602(10,53)0.63	1605(m)	1604(m)vvs	ν(C$_2$-C$_3$)(21)+ν(C$_5$-C$_6$)(17)-ν(C$_1$-C$_6$)(13)	ν(R)8b

					$+\alpha(R)(11)+\beta(C_2-H_7)(7)-\beta(C_6-H_{11})(6)-\nu(C_3-C_4)(6)-\nu(C_1-C_2)(3)$	
1653(7,90)0.13	1653(8,77)0.14	1656(8,27)0.03	1639(m)	1636(ms)vvs	$\nu(C_{15}=C_{17})(67)+\beta_s(=CH_2)(14)-\delta_1(C_{15}-H_{16})(9)-\nu(C_{12}-C_{15})(6)$	$\nu(C=C)$
2867(35,60)0.05	2867(49,20)0.12	2867(44,18)0.10	2870$^{\$}$(m)	2871$^{@}$(ms)	$\nu(C_{20}-H_{23})(45)+\nu(C_{20}-H_{21})(44)+\nu(C_{20}-H_{22})(7)$	$\nu_s(CH_3)a'$
2870(13,100)0.02	2868(7,100)0.02	2868(7,47)0.03	2870$^{\$}$(m)	2871$^{@}$(ms)	$\nu(C_{24}-H_{27})(47)+\nu(C_{24}-H_{25})(42)+\nu(C_{24}-H_{26})(7)$	$\nu_s(CH_3)a'$
2878(11,83)0.03	2878(13,58)0.03	2869(13,100)0.03	2870$^{\$}$(m)	2871$^{@}$(ms)	$\nu(C_{12}-H_{13})(54)+\nu(C_{12}-H_{14})(46)$	$\nu_s(-CH_2)$
2911(5,38)0.73	2909(6,29)0.65	2891(6,24)0.68	-	-	$\nu(C_{12}-H_{14})(54)-\nu(C_{12}-H_{13})(45)$	$\nu_{as}(-CH_2)$
2921(15,35)0.75	2921(17,25)0.75	2921(15,23)0.75	2935(m)	2933 (m)	$\nu(C_{20}-H_{21})(50)-\nu(C_{20}-H_{23})(50)$	$\nu_{as}(CH_3)a''$
2924(16,24)0.75	2922(20,19)0.75	2923(18,15)0.75	2953(m)	-	$\nu(C_{24}-H_{25})(52)-\nu(C_{24}-H_{27})(48)$	$\nu_{as}(CH_3)a''$
2983(6,26)0.21	2984(8,19)0.22	2978(14,46)0.41	2976(m)	2976(m)	$\nu(C_{17}-H_{19})(41)+\nu(C_{17}-H_{18})(32)-\nu(C_{15}-H_{16})(26)$	$\nu_s(=CH_2)$
2991(2,63)0.20	2991(2,47)0.21	2993(7,33)0.52	3001(m)	3002(ms)	$\nu(C_{15}-H_{16})(71)+\nu(C_{17}-H_{19})(23)+\nu(C_{17}-H_{18})(4)$	$\nu(=C-H)$
2993(8,57)0.48	2993(3,25)0.73	2993(13,47)0.41	3001(m)	3002(ms)	$\nu(C_{20}-H_{22})(90)-\nu(C_{20}-H_{23})(4)-\nu(C_{20}-H_{21})(4)$	$\nu_{as}(CH_3)a'$
2994(11,68)0.41	2993(19,72)0.35	2999(3,69)0.16	3001(m)	3002(ms)	$\nu(C_{24}-H_{26})(90)-\nu(C_{24}-H_{25})(4)-\nu(C_{24}-H_{27})(4)$	$\nu_{as}(CH_3)$ a′
3026(5,38)0.36	3033(3,17)0.40	3027(5,23)0.37	-	3039(m)	$\nu(C_6-H_{11})(95)-\nu(C_5-H_{10})(4)$	ν(C-H) 7b
3056(2,13)0.20	3050(4,17)0.24	3051(3,13)0.23	3061(w)	-	$\nu(C_2-H_7)(98)$	ν(C-H)20b
3063(4,58)0.21	3062(4,37)0.20	3064(3,33)0.21	3077(w)	3073(m)	$\nu(C_5-H_{10})(94)+\nu(C_6-H_{11})(4)$	ν(C-H) 2
3066(7,42)0.59	3067(8,32)0.60	3075(7,17)0.54	3077(w)	3073 (m)	$\nu(C_{17}-H_{18})(63)-\nu(C_{17}-H_{19})(35)$	$\nu_{as}(=CH_2)$

*Number before each bracket is frequency (cm^{-1}), numbers within the bracket are relative IR and Raman intensities and number outside bracket is the depolarization ratio, Scaling factor is 0.975 in the frequency range 0-2000 cm^{-1} and 0.956 for the range 3000-3500 cm^{-1} [23].

†Observed frequencies characterised by some notation w=weak band, sh= shoulder, m= medium, ms= medium strong, vs= very strong, s= strong.

#Notations are similar as given in Table -6.

$ 2870 cm^{-1} is the average of the two component IR frequencies 2834 and 2905 cm^{-1};@ 2871 cm^{-1} is the average of the two component Raman frequencies 2934 and 2908 cm^{-1}. The frequency 2870 cm^{-1} corresponds the modes $\nu_s(-CH_2)$, $\nu_s(-^{20}CH_3)$ and $\nu_s(-^{24}CH_3)$. The two components arise due to Fermi resonance between the fundamental mode(s) at 2870 cm^{-1} and the first overtone of the fundamental mode 1439 cm^{-1}, which corresponds to the modes $\beta_s(CH_2)$, $\delta_s(^{20}CH_3)$ and $\delta_s(^{24}CH_3)$

These two frequencies are explained as the combination (165+250=415 cm^{-1}) and overtone (2*279 cm^{-1}) of the fundamental modes of the lowest energy conformer C-I.
